# Protopanaxadiol ginsenoside Rd protects against NMDA receptor-mediated excitotoxicity by attenuating calcineurin-regulated DAPK1 activity

**DOI:** 10.1038/s41598-020-64738-2

**Published:** 2020-05-15

**Authors:** Chen Zhang, Xuedong Liu, Hui Xu, Gengyao Hu, Xiao Zhang, Zhen Xie, Dongyun Feng, Rui Wu, Gang Zhao, Ming Shi

**Affiliations:** 1 Department of Neurology, Xijing Hospital, Fourth Military Medical University, Xi’an, China; 2 Department of Neurology, Characteristic Medical Center of Rocket Force Military, Beijing, China; 3 Institute of Neurosciences, Fourth Military Medical University, Xi’an, China

**Keywords:** Neuroscience, Neurology, Neurological disorders

## Abstract

Neuroprotective strategies in the treatment of stroke have been attracting a great deal of attentions. Our previous clinical and basic studies have demonstrated that protopanaxadiol ginsenoside-Rd (Rd), a monomer compound extracted from *Panax ginseng* or *Panax notoginseng*, has neuroprotective effects against ischemic stroke, probably due to its ability to block Ca^2+^ overload, an usual consequence of the overactivation of NMDA receptor (NMDAR). As an extending study, we explored here whether Rd exerted its neuroprotection as a novel NMDAR blocker. Our whole-cell patch-clamp results showed that Rd reduced NMDAR currents of cultured rat cortical neurons (EC50 = 7.7 μM) dose-dependently by acting on extrasynaptic NMDAR NR2b subunit. However, unexpectedly, cell transfection and radioligand binding assays revealed that Rd did not bind to the NMDAR channel directly. Alternatively, it inhibited the phosphorylation of NR2b at Ser-1303, a target of death associated protein kinase 1 (DAPK1). Moreover, cell-based and cell-free enzymatic assays showed that Rd did not inhibit the activity of DAPK1 directly, but blocked the activity of calcineurin, a key phosphatase for activating DAPK1. Importantly, other protopanaxadiol ginsenosides were also found to have potential inhibitory effects on calcineurin activity. Furthermore, as expected, calcineurin inhibition by cyclosporin A could mimic Rd’s effects and protect against NMDA-, oxygen glucose deprivation- or transient ischemic stroke-induced neuronal injury. Therefore, our present study provided the first evidence that Rd could exert an inhibitive effect on NMDAR-triggered currents and sequential excitotoxicity through mitigation of DAPK1-mediated NR2b phosphorylation by attenuating calcineurin activity.

## Introduction

Ischemic stroke is the leading cause of brain injury, and it has a high morbidity, high mortality and high disability that is seriously threatening public health^1^. Although great efforts have been made to reduce stroke incidence and mortality, current therapeutic strategies for stroke were largely unsuccessful^2^. Until now, the only treatment available is thrombolysis with tissue plasminogen activator (t-PA), but unfortunately, t-PA therapy is extremely limited by its narrow therapeutic window (<4.5 h) and unexpected outcome of hemorrhage^3,4^. Thus, there is a great need for alternative effective and safe treatment of stroke to increase the likelihood that a stroke patient will recover well. Neuroprotective strategies in the treatment of stroke have been attracting a great deal of attentions. A large number of potential neuroprotective agents, which have been showed to be obviously neuroprotective against cerebral ischemic injury in animal models, have proved not to be clinically efficacious^5,6^. To date, only trials of edaravone, a free radical scavenger^7^, NA-1 (Tat-NR2B9c), an inhibitor of postsynaptic density-95 protein^8^ and ginsenoside-Rd^9–11^, a kind of protopanaxadiol ginsenosides extracted from *Panax ginseng* or *Panax notoginseng*, have showed positive results.

*Panax ginseng* and *Panax notoginseng* are two famous traditional herbs popular in China, Korea and Japan. A group of studies have demonstrated that *ginseng* or *notoginseng* has a wide range of pharmaceutical activities in the treatment of cardiovascular disease, cerebrovascular disease, cancer and other diseases^12,13^. Saponins, commonly known as ginsenosides, are the principle ingredients accounting for the efficacy of *Panax ginseng* and *Panax notoginseng*. Currently, more than 150 ginsenosides have been isolated from the ginseng plant, which share a common basic structure, consisting of a gonane steroid nucleus with 17 carbon atoms arranged in four rings. According to the aglycone moieties attached to their core nucleus, ginsenosides are classified into the protopanaxadiol (PPD, including Ra, Rb1, Rb2, Rb3, Rc, Rd, Rg3 and Rh2) and the protopanaxatriol (PPT, including Re, Rf, Rg1, Rg2 and Rh1).

PPD ginsenoside-Rd (Rd) is one of the major ginsenosides in the *Panax ginseng* and *Panax notoginseng*. There is evidence that Rd exerts beneficial effects in a wide range of pathological conditions, including ischemic stroke^14^. A series of our previous clinical and basic studies have demonstrated that Rd could exert a remarkable neuroprotective effect against neuronal injury. In our two randomized, double-blind, placebo-controlled, multicenter trials, Rd showed efficacy and safety for the treatment of acute ischemic stroke^9,10^. In animal models, Rd improved neurological outcome, maintained mitochondrial function, attenuated oxidative damage, and suppressed microglial proteasome-mediated inflammation after transient focal ischemia in rats^11,15–18^. In cultured cells, Rd protected the neurons against hydrogen peroxide- or oxygen-glucose deprivation-induced injury^19,20^. Our recent evidence further showed that Rd protected cultured neurons against NMDA- or glutamate-induced excitotoxicity by inhibiting Ca^2+^ influx^21^, indicating that Rd may act as an blocker of NMDA receptor (NMDAR), one of important receptor-operated Ca^2+^ channels (ROCC), whose overactivation-induced Ca^2+^ overload is the major cause for neuronal excitotoxicity^22–25^. Coincidently, the inhibitory effects of Rd on ROCC were also observed on the vascular smooth muscle cells^26^.

Thus, the evidence above indicated that Rd may be a promising neuroprotectant with distinctive advantages for the treatment of ischemic stroke, and the mechanism may involve its potential capability of inhibiting of NMDAR. As an extending study, here we explored whether Rd could serve as an NMDAR blocker to act against neuronal excitotoxicity.

## Materials and Methods

### Materials

Ginsenosides Rd, Rb1, Rb2, Rg1, Rg2, Rg3, and Rh2 were obtained from Tai-He Biopharmaceutical Co. Ltd. (Guangzhou, China), and relative information was summarized in Supplementary Table 1. Stock solutions of ginsenosides were prepared in saline containing 10% propanediol (v/v). 4-AP, AP-5, bicuculline, CNQX, FK506, glycine, Gö6983, nifedipine, NMDA, and NVP-AAM077 were purchased from Sigma-Aldrich (St. Louis, MO, USA). Ifenprodil and TTX were obtained from Tocris Bioscience (Ellisville. Mo, UK). CK59, DAPK inhibitor (DAPKi, (4Z)-4-(3-Pyridylmethylene)-2-styryl-oxazol-5-one), and PP2 were from Merck (Darmstadt, Germany). Cyclosporin A (CsA) was from Abcam (Cambridge, UK). Stock solutions of 4-AP, AP-5, bicuculline, glycine, ifenprodil, NMDA, NVP-AAM077, and TTX were prepared in distilled water, and those of CK59, CNQX, CsA, DAPKi, FK506, Gö6983, nifedipine, and PP2 were prepared in DMSO. All these drugs were diluted to their final concentrations in the corresponding extracellular solution just before each experiment. The final concentration of DMSO was maintained at ≤0.1%.

### Cell culture

For primary culture of cortical neurons, the cells were prepared from embryonic day 18 Sprague Dawley (SD) rats, as previously described^27^. The neurons were cultured in Neurobasal medium (Gibco, Grand Island, NY, USA) supplemented with 2% B27 (Gibco) and 0.5 mM L-glutamine (Sigma), fed every 3–4 days with fresh media, and used after 10–14 days *in vitro*. The purity of the culture was more than 95% as determined by specific immunostaining of Tuj1 (Cat. No. ab68193, Abcam). For culture of HEK293 cells, the cells were cultured in Dulbecco’s modified Eagle’s medium (DMEM) supplemented with 10% fetal bovine serum, 100 U/ml of penicillin, and 100 μg/ml of streptomycin at 37 °C in a humidified atmosphere of 5% CO_2_.

### Cell transfection

HEK293 cells were seeded into six-well plates at a density of 0.5 × 10^6^ cells/cm^2^. After 18 h, the cells were transfected with 2 μg of pCAG-NR1 plasmid and 2 μg of pCAG-NR2a or 2 μg of pCAG-NR2b plasmid per well using Lipofectamine 2000 reagent (Invitrogen, Eugene, OR, USA). Six hours after transfection, cells were supplemented with fresh culture medium (1 ml/well). 24–48 h after transfection, HEK293 cells were used for western blot analysis or electrophysiological experiments. pCAG-NR1, pCAG-NR2a, and pCAG-NR2b plasmids were kindly gifted from Prof. Jian-Hong Luo (Zhejiang University School of Medicine, China).

### Electrophysiological recordings

Whole-cell patch-clamp recordings were performed on rat cortical neurons or transfected HEK293 cells as previously described^28^. The extracellular solution (ECS) contained the following (in mM): 140 NaCl, 5 KCl, 2.5 CaCl_2_, 1 MgCl_2_, 10 HEPES and 10 D-glucose, titrated to pH 7.3 with NaOH and osmolarity 310–320 mOsm. The composition of intracellular solution (in mM) was 120 K-Gluconate, 5 NaCl, 1 MgCl_2_, 0.2 EGTA, 2 MgATP, 0.1 Na_2_GTP, 10 HEPES and 10 Phosphocreatine disodium (pH 7.2; 295–310 mOsm). Patch pipettes (3–5 MΩ) were pulled from borosilicate glass capillaries using a programmed micropipette puller (P-97, Sutter Instruments, CA, USA). Whole-cell recordings were obtained using an Axopatch 200 A amplifier (Molecular Devices, Sunnyvale, CA). Neurons or HEK293 cells with resting membrane potential more negative than −50 mV were selected for further experiments. The series resistance was less than 30 MΩ and monitored throughout recordings. The recording was discarded if series resistance varied by more than 10%. All recordings were filtered at 2 kHz, sampled at 10 kHz and stored using Clampfit (v10.0, Molecular Devices, Sunnyvale, CA, USA. https://www.moleculardevices.com)

For NMDAR-mediated current recordings, the cells were perfused with Mg^2+^-free ECS, which contains the following (in mM): 140 NaCl, 5 KCl, 2.5 CaCl_2_, 10 HEPES and 10 glucose. The NMDAR currents were evoked by 100 μM NMDA and 10 μM glycine in Mg^2+^-free ECS with 1 μM TTX using “Y-tube” perfusion system^29–31^. The cell membrane was held at −60 mV. For excitotoxicity study, the neurons were first treated with 100 μM NMDA and 10 μM glycine for 30 min to induce excitotoxicity, and then NMDAR currents were recorded as the above description.

For NMDAR-mediated spontaneous miniature excitatory postsynaptic current (NMDAR-mEPSCs) recordings, the neurons were perfused with Mg^2+^-free ECS in the presence of 1 μM TTX, 10 μM bicuculline, 10 μM CNQX and 10 μM nifedipine. The NMDAR-mEPSCs were recorded at a holding membrane potential of −60 mM in gap-free mode under voltage-clamped configuration. Synaptic events before and after Rd application were recorded for 10–20 min to acquire sufficient data and analyzed using Mini Analysis Program (v6.0, Synaptosoft, Decatur, GA, http://www.synaptosoft.com/MiniAnalysis).

For extrasynaptic NMDAR current recordings, which were performed according to MK-801 trapping protocol as described previously^32,33^. Briefly, the neurons were perfused with Mg^2+^-free ECS in the presence of 30 μM bicuculline, 2 mM 4-AP and 10 μM nifedipine to increase activation of synaptic NMDAR. Then, 20 μM MK-801 was added to irreversibly block the activated synaptic NMDAR channels. After MK-801 washout, the extrasynaptic NMDAR currents were elicited by 100 μM NMDA and 10 μM glycine in Mg^2+^-free ECS with 1 μM TTX, 10 μM bicuculline, 10 μM CNQX and 10 μM nifedipine using “Y-tube” perfusion system.

For NMDAR current analysis, amplitudes of peak NMDAR currents (I_NMDA_) and normalized I_NMDA_ were evaluated. Normalized I_NMDA_ = (I_NMDA_ after drug application)/(I_NMDA_ before drug application) × 100%. To generate the concentration-response curve, the data were fitted with the equation^34^: I_R_/I_max_ = 1/[1 + (C/EC_50_)^H^], in which I_R_ is the difference between before and after Rd treatment, I_max_ is the maximal response in the absence of Rd, I_R_/I_max_ is the percentage of reduction, C is the concentration of Rd, H is the Hill coefficient, and EC_50_ is a concentration of Rd, producing half maximal effect on NMDAR currents. The data from electrophysiological recordings were plotted by OriginPro (v8.0, OriginLab, Northampton, MA, USA. https://www.originlab.com).

### ***In vitro*and*in vivo*ischemic injury models**

For *in vitro* injury model, cultured neurons were subjected to oxygen glucose deprivation (OGD) according to our previous study^20^. At the end of OGD insult, corresponding drugs were added. Twenty-four hours later, Western blotting (as described below) was performed for detection of various proteins and TUNEL staining was for evaluation of neuronal apoptosis, which was carried out as described by the manufacturer (Roche, Indianapolis, IN, USA) following by nucleus staining with Hoechst 33342 (1 μg/ml, Sigma).

For *in vivo* injury model, a total of 60 adult male SD rats (280–300 g, from the animal center of the Fourth Military Medical University) were subjected to middle cerebral artery occlusion (MCAO) as described previously^15–17,35^. Briefly, after the rats were anesthetized with 4% isoflurane in 70% N_2_O/30% O_2_ using a mask, a 4–0 nylon suture coated with poly-L-lysine was applied to occlude the origin of right MCA. After 2 h of occlusion, the suture was removed to restore the blood flow. During and after operation, regional cerebral blood flow was monitored by a laser Doppler flowmetry (PeriFlux 5000, Perimed AB, Sweden) and rectal temperature was maintained at 37.5 °C by using a feedback-controlled heating pad. The rat was suspended by the tail and left forelimb flexion was defined as a completed stroke model. Immediately after MCAO insult, Rd (10 mg/kg) or/and CsA (10 mg/kg) were applied intraperitoneally. For evaluation of infarct volume and animal neurological function 24 h after MCAO, TTC stain and behavioral tests were carried out respectively according to our previous studies^15^. At 24 h post-MCAO, brain tissues containing infarct and penumbra were collected for Western blot, as described below. For each set of comparison, at least five experimental and control rats were included. The animal experiment procedures were approved by the Animal Care and Use Committee of Fourth Military Medical University and were in compliance with the Guidelines for the Care and Use of Laboratory Animals.

### Western blot analysis

Immunoblotting assays were performed as described previously^11,17,20^. In brief, the cultured cells and rat brain tissues were collected, and total proteins were extracted using RIPA lysis buffer (PBS, 1% NP40, 0.5% sodium deoxycholate, 0.1% SDS, 0.25 mm PMSF, 5 mg/ml aprotinin, 1 mm sodium orthovanadate). After electrophoresed on 10% SDS-polyacrylamide gels, proteins were transferred onto nitrocellulose membranes, which were incubated at 4 °C overnight with following antibodies: β-actin (Cat. No. sc-47778. Santa Cruz Biotechnology, Santa Cruz, CA, USA), anti-AKT (Cat. No. 2920. Cell Signaling Technology, Danvers, MA, USA), anti-phospho-AKT (Cat. No. 4058. Cell Signaling Technology), anti-cleaved caspase3 (Cat. No. 9664. Cell Signaling Technology), anti-DAPK1 (Cat. No. D1319. Sigma), anti-phospho-DAPK (Cat. No. D4941. Sigma), anti-p44/42 MAPK (ERK1/2, Cat. No. 4695. Cell Signaling Technology), anti-phospho-ERK1/2 (Cat. No. 4370. Cell Signaling Technology), anti-GAPDH (Cat. No. sc-47724. Santa Cruz Biotechnology), anti-NMDAR1 (NR1, Cat. No. ab109182. Abcam), anti-NMDAR2a (NR2a, Cat. No. ab14596. Abcam), anti-NMDAR2b (NR2b, Cat. No. ab65783. Abcam), anti-phospho-NR2b (Ser1303, Cat. No. 07–398. Millipore, Darmstadt, Germany), anti-phospho-NR2b (Ser1480, Cat. No. ab73014. Abcam), or anti-phospho-NR2b (Tyr1472, Cat. No. 4208. Cell Signaling Technology). For detection, horseradish peroxidase-conjugated secondary antibodies (Cat. No. 7076 and 7074. Cell Signaling Technology). Five independent experiments were performed. The protein bands were visualized with an Enhanced Chemiluminescence System (Amersham Biosciences, Piscataway NJ, USA) were used. All band signals were quantified using ImageJ (v1.43, NIH software, Bethesda, MD, USA, http://imagej.nih.gov/ij) and the data acquired were normalized to β-actin or GAPDH expression and further normalized to the controls. The data from immunoblotting assays were plotted by OriginPro (v8.0, OriginLab).

### Radioligand-receptor binding assay

Ginsenosides used for radioligand binding assay were listed in Supplementary Table 1. Each chemical was dissolved in DMSO at a concentration of 10 mM. The NMDAR binding assay was performed as described previously^36^. In brief, membrane containing NMDAR was prepared from rat cerebral cortex. The membrane fraction (containing 24 μg of protein) was incubated with 10 nM [^3^H]CGP39653 (40.5 Ci/mmol; PerkinElmer Life and Analytical Sciences, Waltham, MA), a NMDA antagonist radioligand^37^, for 30 min at 25 °C in the absence or presence of ginsenosides. Retained radioactivity was counted by a TopCount (PerkinElmer Life and Analytical Sciences). Results showing an inhibition higher than 50%, between 25% and 50%, and lower than 25% are considered to represent significant, weak to moderate, and no significant effects of the test ginsenosides, respectively.

### Detection of DAPK and calcineurin activities

Cell-based or cell-free enzymatic assays for detecting DAPK1 and calcineurin activities were performed according to the instructions of commercial kits. ADP-Glo & DAPK1 Kinase Enzyme System were from Promega (Madison, WI, USA). Cellular Calcineurin Phosphatase Activity Assay kit was from Abcam. After different concentrations of Rd (0.1, 1, 10, 100 μM) were used, the levels of phosphated substrate (p-MBP) and released phosphate were examined to indicate the activities of DAPK1 and calcineurin, respectively. All the data acquired were normalized to the controls.

### Data analysis

All data were represented as mean ± SEM. Student’s t-test and paired t-test were used for comparison between two groups, and on-way ANOVA followed by Tukey test for comparison among multiple groups. The software SPSS 15.0 was used to analyze all the data, and statistical significance was defined as *p* < 0.05.

## Results

### Rd reduces NMDAR currents in cultured neurons

**Figure 1:**
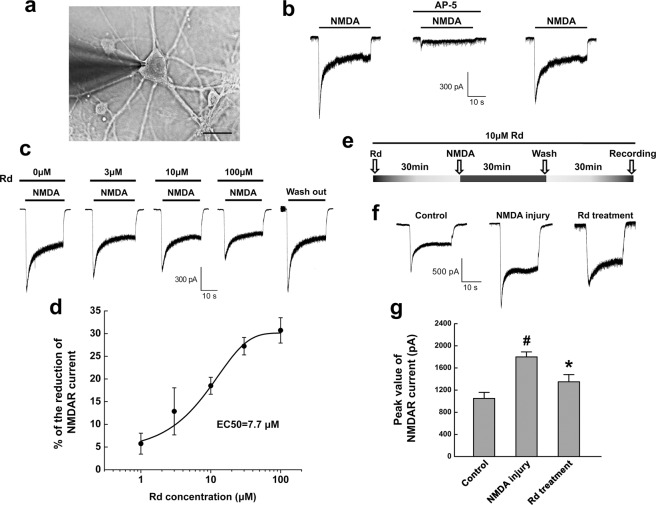
Rd reduces NMDAR-mediated currents in cultured neurons. (**a**) Photomicrographs showed the representative cultured neuron, which was chosen for whole-cell patch-clamp recording. Scale bar=20 μm. (**b**) A typical inward NMDAR current (left) was evoked in the presence of NMDA (100 μM), which was remarkably attenuated by AP-5 (50 μM) (middle). After AP-5 washout, the magnitude of NMDAR currents was recovered near to that before AP-5 application (right). (**c**) Rd with different concentrations (0 μM, 3 μM, 10 μM, 100 μM) reduced NMDA-evoked currents. 0 μM Rd refers to the control only received with the same volume of Rd’s solvent. (**d**) The reduction percentages of peak value of NMDAR currents in the presence of Rd in different concentrations (1 μM, 3 μM, 10 μM, 30 μM, 100 μM). (**e**) The diagram showed the protocol for cultured neurons subjected to NMDA injury. (**f**) After NMDA injury, the peak values of NMDAR currents were elevated compared with the control (regular NMDAR current as indicated in (**b**)), which could be markedly decreased by Rd (10 μM) addition. (**g**) Quantitation of the peak values of NMDAR currents in different groups. ^#^ *p* < 0.05 vs. the control; ^*^p < 0.05 vs. NMDA injury group. Error bars = S.E.M.

To explore whether NMDAR is a potential target of Rd, we examined the effects of Rd on NMDAR currents of cultured rat cortical neurons (Fig. 1a). The rapid and brief application of NMDA (100 μM) elicited inward currents that partially desensitized within 5 s to steady state. The inward currents can be completely inhibited by NMDAR antagonist AP-5 (50 μM), and were recovered when AP-5 was washed off (Fig. 1b). These results were consistent with previous reports^34,38,39^, indicating that the evoked currents were typical NMDAR-mediated currents. After application of Rd at different concentrations (1 μM, 3 μM, 10 μM, 30 μM, 100 μM), compared with the control, in which no Rd (0 μM) but only same volume of Rd’s solvent was added, Rd could reduce the peak amplitudes of NMDAR currents in a dose-dependent manner (1 μM: 5.7 ± 2.3%, n = 20; 3 μM: 12.9 ± 5.2%, n = 20; 10 μM: 18.5 ± 1.9%, n = 20; 30 μM: 27.2 ± 1.9%, n = 20; 100 μM: 30.7 ± 2.8%, n = 20), and the EC_50_ was 7.7 μM. When Rd washout, NMDAR currents recovered to the amplitude similar to that of the control (Fig. 1c,d).

Next, we investigated whether Rd affected NMDAR currents of the neurons under excitotoxic injury. The neurons were induced to excitotoxicity with application of 100 μM NMDA and 10 μM glycine for 30 min as described previously (Fig. 1e)^33^. After NMDA injury, the peak values of NMDAR currents (−1875.0 ± 67.5 pA, n = 20) were significantly increased compared to the control (−1047.3 ± 102.5 pA, n = 20) while 10 μM Rd decreased injury-induced elevated NMDAR currents to −1330.2 ± 139.1 pA (n = 20, *p* = 0.064, Fig. 1f,g). It was noted that in spite of Rd treatment, the extent of reduction of NMDAR currents in excitotoxic neurons (29.1 ± 3.1%) was still more than that of control neurons (18.5 ± 1.9%). Thus, these results suggested that Rd could moderately attenuate the activation of NMDAR channels under normal and excitotoxic conditions.

### Rd selectively acts on extrasynaptic NR2b subunits

**Figure 2:**
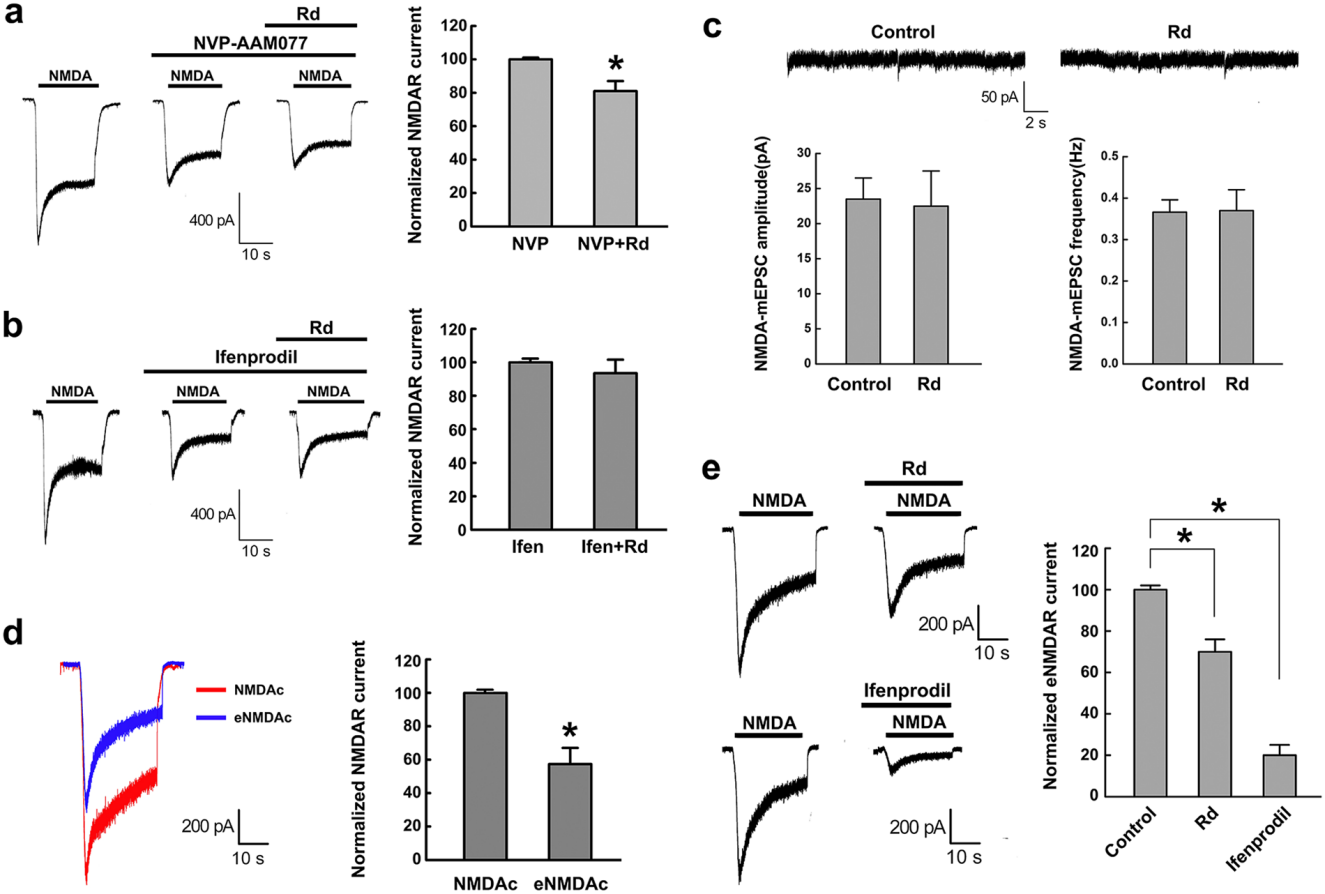
Rd selectively acts on extrasynaptic NMDAR NR2b subunits. (**a,b**) It was NR2b antagonist ifenprodil (**b**) but not NR2a antagonist NVP-AAM077 (**a**) that affected Rd-induced reduction of NMDAR currents. *p < 0.05 vs. NVP group. (**c**) Before (the control) and after 10 μM Rd application, both amplitudes and frequencies of NMDAR-mEPSCs were not changed. (**d**) A representative trace showed the whole NMDAR current (NMDAc, red) and extrasynaptic NMDAR current (eNMDAc, blue). *p < 0.05, vs. NMDAc. (**e**) The magnitudes of eNMDAc were reduced after application of either 10 μM Rd (top) or 10 μM ifenprodil (bottom), compared with their respective controls only treated with the same volumes of solvents. *p < 0.05, vs. the control. Error bars = S.E.M.

Given that NMDAR consists of an essential NR1 subunit and one or more regulatory NR2 subunits (NR2a-b), among which NR2a- and NR2b-containing NMDAR are considered as the main types of functional NMDAR channels in CNS neurons^40^, we next investigated which NMDAR subunits Rd may actually act on by using NR2a and NR2b antagonists. Both NR2a antagonist NVP-AAM077 (NVP, 0.5 μM) and NR2b antagonist ifenprodil (10 μM) decreased NMDAR currents (NVP: 36.0 ± 1.7%, n = 20; ifenprodil: 54.0 ± 1.2%, n = 20) as compared with NMDAR treatment alone. Subsequent application of Rd (10 μM) further reduced NVP-induced decreased NMDAR currents (18.2 ± 5.2%, n = 20), indicating that NVP did not affect the inhibitory effect of Rd on NMDAR currents. By contrast, Rd failed to suppress ifenprodil-induced decreased NMDAR currents, indicating that ifenprodil could eliminate the inhibitory effect of Rd on NMDAR currents (Fig. 2a,b). These results implied that Rd probably acted on NR2b but not NR2a subunit to affect NMDAR currents.

A growing body of evidence has suggested that NR2b subunit predominantly localizes at extrasynaptic sites and may have a cell death-inducing role after injury^41–44^. To explore whether Rd could selectively act on extrasynaptic NR2b, we compared the changes in synaptic and extrasynaptic NMDAR currents in the presence of Rd (10 μM). Our results showed that Rd did not affect the amplitude and frequency of NMDAR-mEPSCs (n = 20), which represent the activity of synaptic NMDARs (Fig. 2c). Then, using a classic MK-801 trapping protocol^32,33^ to record the extrasynaptic NMDAR currents (eNMDAc) by blocking synaptic NMDAR channels, we showed a representative trace of eNMDAc with an amplitude of about 58.2 ± 8.2% (n = 20) to the whole NMDA-evoked currents (Fig. 2d). The peak amplitudes of eNMDAc were significantly decreased to 70.5 ± 6.3% (n = 20) and 20.6 ± 5.0% (n = 20) after application of 10 μM Rd and ifenprodil, respectively (Fig. 2e). Therefore, our results above indicated that Rd may selectively act on extrasynaptic NR2B subunits.

### Rd acts on NMDAR in an indirect way

**Figure 3:**
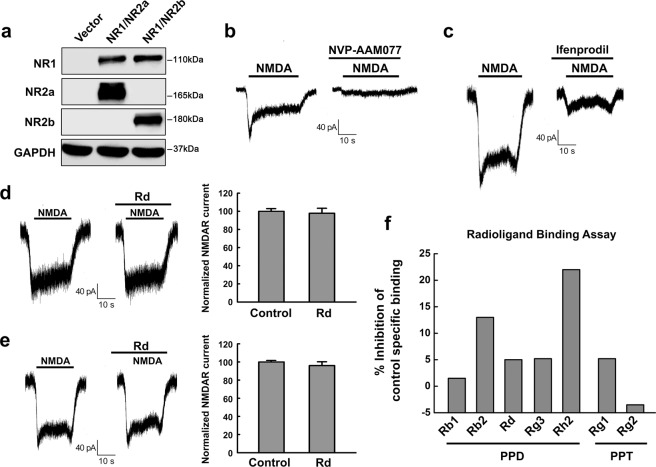
Rd does not bind to NMDAR directly. (**a**) Western blotting results showed successful co-transfection of recombinant NR1/NR2a or NR1/NR2b in HEK293 cells. GAPDH was used as an internal control. For comparison easily, shown blots were cropped, and the full-length blots were presented in Supplementary Fig. 6. (**b,c**) NMNA-evoked currents in HEK293 cells co-transfected with NR1/NR2a and NR1/NR2b were eliminated by applying NVP-AAM077 (**b**) and ifenprodil (**c**), respectively. (**d,e**) Rd did not affect NMNA-evoked currents in HEK293 cells co-transfected with NR1/NR2a (d) or NR1/NR2b (**e**). (**f**) The inhibition percentages of [^3^H]CGP39653 specific binding to NMDAR in the presence of PPD ginsenosides (Rb1, Rb2, Rd, Rg3, and Rh2) and PPT ginsenosides (Rg1 and Rg2) were revealed by radioligand binding assay. Error bars = S.E.M.

To further determine whether Rd may directly bind to NMDAR to exert its inhibitory effect on NMDAR currents, we performed cell transfection and radioligand-receptor binding assays. By co-transfecting HEK293 cells, which do not express NR1, NR2a and NR2b in themselves, with NR1 and NR2a or NR2b plasmids, we ecto-expressed NMDAR subunits in HEK293 cells. Western blot analysis confirmed successful expression of NR1/NR2a or NR1/NR2b subunits in HEK293 cells (Fig. 3a). Displayed blots were cropped, and full-length blots were presented in Supplementary Fig. 6. Whole-cell patch recording showed that NMDA could evoke NMDAR currents of transfected HEK293 cells expressing NR1/NR2a or NR1/NR2b subunits, which can be blocked by NVP and ifenprodil, respectively (Fig. 3b,c). Unexpectedly, however, application of 10 μM Rd seemingly did not affect NMDAR currents of HEK293 cells expressing either NR1/NR2a or NR1/NR2b receptors (n = 20 cells each group) (Fig. 3d,e), suggesting that Rd, unlike classic NMDAR antagonists, may inhibit NMDAR currents through an indirect effect on NMDAR.

To verified the results of cell transfection assay, we performed a radioligand binding experiment using the selective NMDA antagonist radioligand [^3^H]CGP39653^36,37^ to test whether Rd was able to bind to NMDAR directly. Binding assay showed that Rd with the concentration of 10 μM inhibited only 5.2% of [^3^H]CGP39653 specific binding, and this displacement value was much lower than 25% cutoff, which was not considered significant (Fig. 3f), indicating that Rd had little affinity binding to NMDAR. At the same time, we tested other PPD ginsenosides (including Rb1, Rb2, Rg3, Rh2) and PPT ginsenosides (including Rg1, Rg2) as well, and found that all the displacement values were lower than 25% cutoff (Fig. 3f). Taken together, the negative results of transfection and binding experiments revealed that Rd was not able to bind to NMDAR directly, implying that it may act on NMDAR in an indirect way.

### Rd inhibits NR2b phosphorylation at Ser-1303

**Figure 4:**
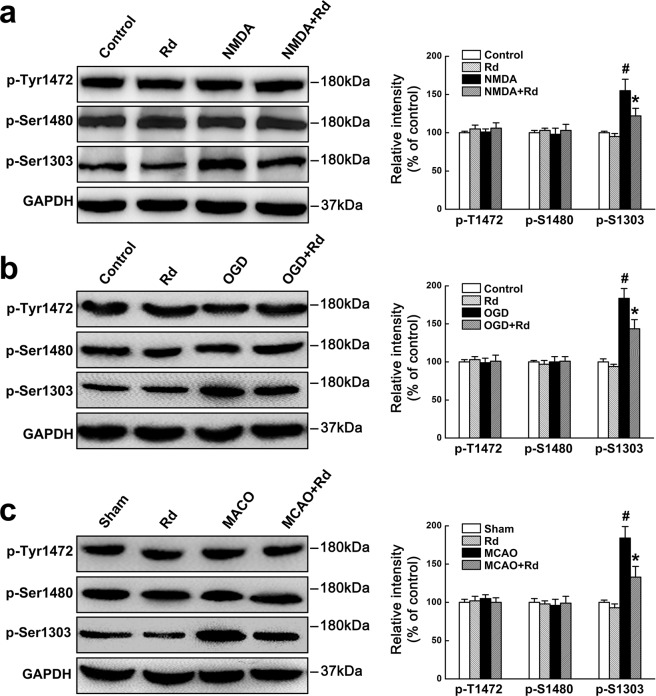
Rd inhibits NMDAR NR2b phosphorylation at Ser-1303 after injuries. (**a–c**) Western blotting analysis showed that in NMDA- (**a**) or OGD-injured (**b**) cultured neurons and MCAO-injured rat brains (**c**), the levels of NR2b phosphorylation at Ser-1303 but not at Tyr1472 and Ser1480 sites were increased, which was attenuated by Rd treatment markedly. GAPDH was used as an internal control. For comparison easily, shown blots were cropped, and the full-length blots were presented in Supplementary Fig. 7. ^#^*p* < 0.05 vs. the control or the sham group; *p < 0.05 vs. NMDA, OGD, or MCAO injury group. Error bars = S.E.M.

Various factors are involved in modulating NMDAR functions, among which phosphorylation is one of important ways. NMDAR NR2b subunit has long intracellular C-terminal domain containing several key phosphorylation sites, which are essential for the functional regulation of NMDAR^45–48^. Then we investigated whether Rd could affect the phosphorylation of NR2b subunit, mainly focusing on Ser1303, Tyr1472, and Ser1480 sites. Cultured neurons were subjected to NMDA- or OGD-induced injury in the presence or absence of Rd. Immunoblotting results showed that the expression of NR1, NR2a or NR2b was not changed after injury (Supplementary Fig. 1, and data not shown). Displayed blots were cropped, and full-length blots were presented in Supplementary Fig.11. However, NMDA- or OGD-induced injury increased the levels of phosphorylation of NR2B at Ser1303 (p-Ser1303), but not Tyr1472 (p-Tyr1472) or Ser1480 (p-Ser1480). Importantly, elevated level of p-Ser1303 can be attenuated by Rd significantly, compared with the controls. Similarly, in a rat transient cerebral ischemic model, Rd (10 mg/kg, i.p.) decreased MCAO-induced increased level of p-Ser1303. It was noted that Rd did not affect NR2b phosphorylation in the control neurons and sham rats (Fig. 4a–c). Displayed blots were cropped, and full-length blots were presented in Supplementary Fig. 7. Thus, these results indicated that Rd may affect the phosphorylation of NR2B at Ser1303 to interfere NMDAR functions.

### Rd affects NR2b phosphorylation by regulating DAPK activity

**Figure 5:**
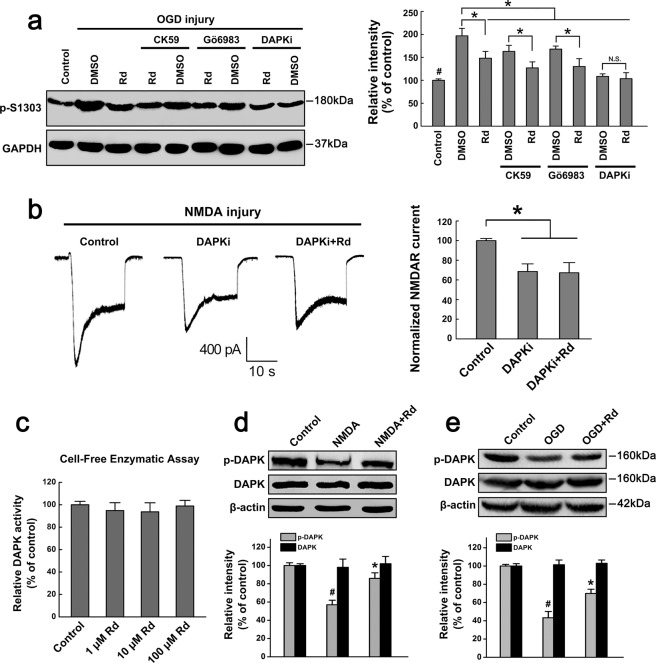
Rd acts on DAPK to affect NMDAR phosphorylation and currents. (**a**) After cultured neurons were subjected to OGD injury, Western blotting analysis showed that CaMKII antagonist CK59, PKC antagonist Gö6983, and DAPK inhibitor DAPKi attenuated the elevated levels of p-Ser1303 but only DAPKi blocked the inhibitory effects of Rd on NR2b phosphorylation at Ser1303. Neurons in the control group were received the same quantity of DMSO and propanediol. GAPDH was used as an internal control. Shown blots were cropped to see easy, and the full-length blots were presented in Supplementary Fig. 8. ^#^*p* < 0.05 vs. the control; *p < 0.05 vs. the DMSO groups; N.S., no significance. (**b**) whole-cell patch results showed that after NMDA injury, DAPKi blocked the inhibitory effects of Rd on NMDAR currents. *p < 0.05 vs. the control. (**c**) Cell-free enzymatic assay showed that Rd did not affect DAPK activity. (**d,e**) NMDA- (**d**) or OGD-induced (**e**) injury decreased the levels of DAPK phosphorylation, and however, Rd promoted the phosphorylation of DAPK. β-actin was used as an internal control. For comparison easily, shown blots were cropped, and the full-length blots were presented in Supplementary Fig. 8. ^#^*p* < 0.05 vs. the control; *p < 0.05 vs. NMDA, or OGD injury group. Error bars = S.E.M.

Since serine/threonine phosphorylation is mainly catalyzed by CaMKII or PKC^48^, we then examined whether Rd affected Ser1303 phosphorylation of NR2B by acting on these kinases. Using CaMKII antagonist CK59 (10 μM) and PKC antagonist Gö6983 (1 μM), we found that CK59 or Gö6983 alone could attenuated the elevated levels of p-Ser1303 in OGD-injured neurons to some extent, and however, both of them did not block the inhibitory effects of Rd on p-Ser1303 levels (Fig. 5a). Displayed blots were cropped, and full-length blots were presented in Supplementary Fig. 8. Consistently, whole-cell patch results showed that CK59 or Gö6983 did not affect Rd-induced reduction of NMDAR currents in NMDA-injured neurons (CK59: 16.1 ± 5.3%, n = 20, *p* < 0.05; Gö6983: 14.5 ± 3.5%, n = 20, *p* < 0.05), as compared with the controls (Supplementary Fig. 2). These results indicated that Rd may not act on CaMKII or PKC to affect NR2b phosphorylation.

A recent study identified the death associated protein kinase 1 (DAPK1) as a specific kinase for NR2b phosphorylation at Ser1303^33^. These results prompted us to investigate whether Rd could act on DAPK1. Using a specific DAPK inhibitor DAPKi (10 μM), we found that it not only decreased OGD-elevated levels of p-Ser1303 but also block the inhibitory effects of Rd on p-Ser1303 expression (Fig. 5a). Consistently, whole-cell currents recorded from NMDA-injured neurons showed that DAPKi alone and together with Rd decreased the peak amplitude of NMDAR currents from −1810.8 ± 78.6 pA (n = 20) to −1238.7 ± 63.5 pA (n = 20) and −1208.6 ± 68.2 pA (n = 20), respectively (Fig. 5b), indicating that inhibitory effect of Rd on NMDAR currents could be eliminated by DAPKi. These results implied that Rd may act on DAPK1 and subsequently affect NR2b phosphorylation and sequential NMDAR channel conductance.

To explore whether Rd may directly affect DAPK1, we applied an *in vitro* cell-free enzymatic assays, in which active DAPK1 was incubated with its substrate MBP in the presence or absence of different concentrations of Rd, and the product of p-MBP serves an index for DAPK1 activity. Unexpectedly, the results showed that 1–100 μM Rd did not affect DAPK1 activity compared with the control (Fig. 5c), suggesting that Rd may not act on DAPK1 directly. Given that DAPK activity is mainly determined by its phosphorylation state, that is, phosphorylation and dephosphorylation represent inactivated and activated forms of DAPK1, respectively^49^, we then tested the effects of Rd on DAPK1 phosphorylation (p-DAPK). Western blotting results showed that in both NMDA- and OGD-injured neurons, the levels of p-DAPK were significantly decreased compared with the controls. Rd did not affect the total expression of DAPK, but did elevate NMDA- and OGD-induced decreased levels of p-DAPK (Fig. 5d,e). Similar results were also observed in rats subjected to MCAO insults (Supplementary Fig. 3). Displayed blots were cropped, and full-length blots were presented in Supplementary Figs 8 and 12, respectively. These results indicated that Rd promoted DAPK phosphorylation to inhibit its activity, and subsequently decreased NR2b phosphorylation at Ser1303.

### Rd acts on calcineurin to regulate DAPK activity

**Figure 6:**
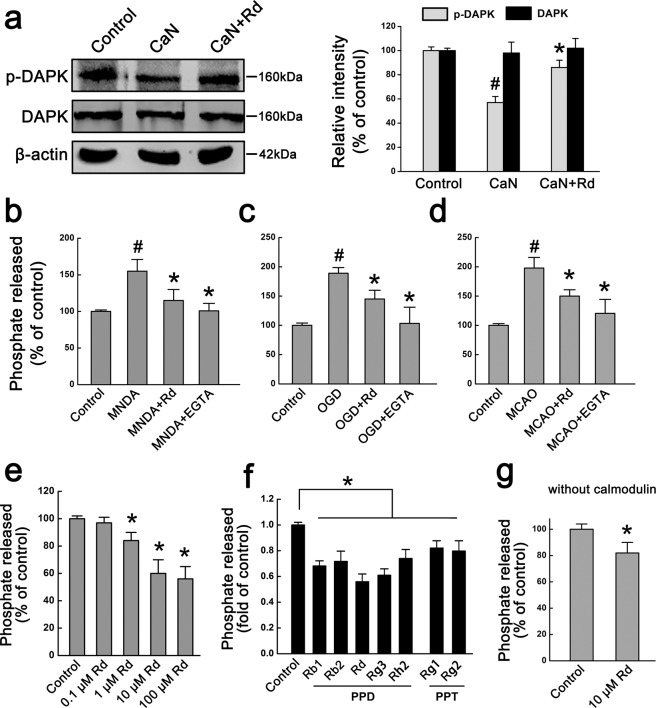
Rd directly acts on calcineurin to affect DAPK activity. (**a**) Cell-based enzymatic assay showed that purified calcineurin (CaN) significantly reduced the level of p-DAPK whereas Rd was able to counteract the effects of calcineurin. β-actin was used as an internal control. For comparison easily, shown blots were cropped, and the full-length blots were presented in Supplementary Fig. 9. ^#^*p* < 0.05 vs. the control; *p < 0.05 vs. CaN treatment group. (**b–d**) In NMDA- or OGD-injured neurons, and MCAO-injured rat brains, the percentages of phosphate released were markedly increased, which however, was attenuated by the treatment of Rd or calcium chelator EGTA. ^#^*p* < 0.05 vs. the controls; *p < 0.05 vs. NMDA, OGD or MCAO injury group. (**e**) Cell-free enzymatic assay showed that Rd dose-dependently inhibited calcineurin activity. (f) PPD (Rb1, Rb2, Rg3, Rh2) and PPT ginsenosides (Rg1, Rg2) had potential inhibitory effects on calcineurin activity, as revealed by cell-free enzymatic assay. (**g**) Rd inhibited calcineurin activity even in the absence of calmodulin. *p < 0.05 vs. the control. Error bars = S.E.M.

Since DAPK phosphorylation is regulated by calcineurin, a Ca^2+^/calmodulin-dependent serine phosphatase^49,50^, we tested whether Rd could act on calcineurin. Using a cell-based enzymatic assay, in which purified recombinant calcineurin enzyme was added to the proteins extracted from normal neurons, and then the levels of p-DAPK were examined by immunoblotting assay. Compared with the control, calcineurin application reduced the level of p-DAPK significantly while Rd could attenuate calcineurin-induced decreased level of p-DAPK but did not affect the total expression of DAPK (Fig. 6a), suggesting that Rd may inhibit calcineurin activity. Displayed blots were cropped, and full-length blots were presented in Supplementary Fig. 9. To further explore the changes in calcineurin activity and confirm the effects of Rd on calcineurin activity after injury, we prepared the proteins extracted from NMDA- or OGD-injured neurons, and MCAO-injured rat brain tissues, and incubated these proteins with RII phosphopeptide (a calcineurin substrate). Free phosphate released was detected as a commercial kit described to indicate calcineurin activity. The results showed that compared with the control, calcineurin activity was markedly enhanced by NMDA, OGD, or MCAO insult, which could be significantly attenuated by the treatment of Rd or EGTA, a calcium chelator (Fig. 6b–d).

To test whether Rd inhibited calcineurin activity directly, we performed a cell-free enzymatic assay, in which calcineurin enzyme incubated with its substrate in the presence or absence of different concentrations of Rd. The results showed that Rd inhibited calcineurin activity in a dose-dependent manner (Fig. 6e), as compared with the control (0.1 μM: 97.0 ± 4.5%, *p* > 0.05; 1 μM: 84.4 ± 6.3%, *p* < 0.05; 10 μM: 63.1 ± 9.8%, *p* < 0.05; 100 μM: 58.6 ± 8.5%, *p* < 0.05). In addition, we also examined other PPD ginsenosides (including Rb1, Rb2, Rg3, Rh2) and PPT ginsenosides (including Rg1, Rg2), and found that PPD had more potential inhibitory effects on calcineurin activity than PPT, among which Rd’s effect was most prominent (Fig. 6f). Additionally, we found that even omission of calmodulin in this cell-free enzymatic system, Rd could still inhibit calcineurin activity to an extent (81.6 ± 9.8%) just slighter than that with calmodulin (59.5 ± 10.5%) (Fig. 6g). Taken together, these results revealed that Rd may directly inhibit calcineurin activity, subsequently enhancing the phosphorylation of DAPK and inhibiting its activity.

### Rd and calcineurin inhibitor CsA protect against neuronal injury

**Figure 7:**
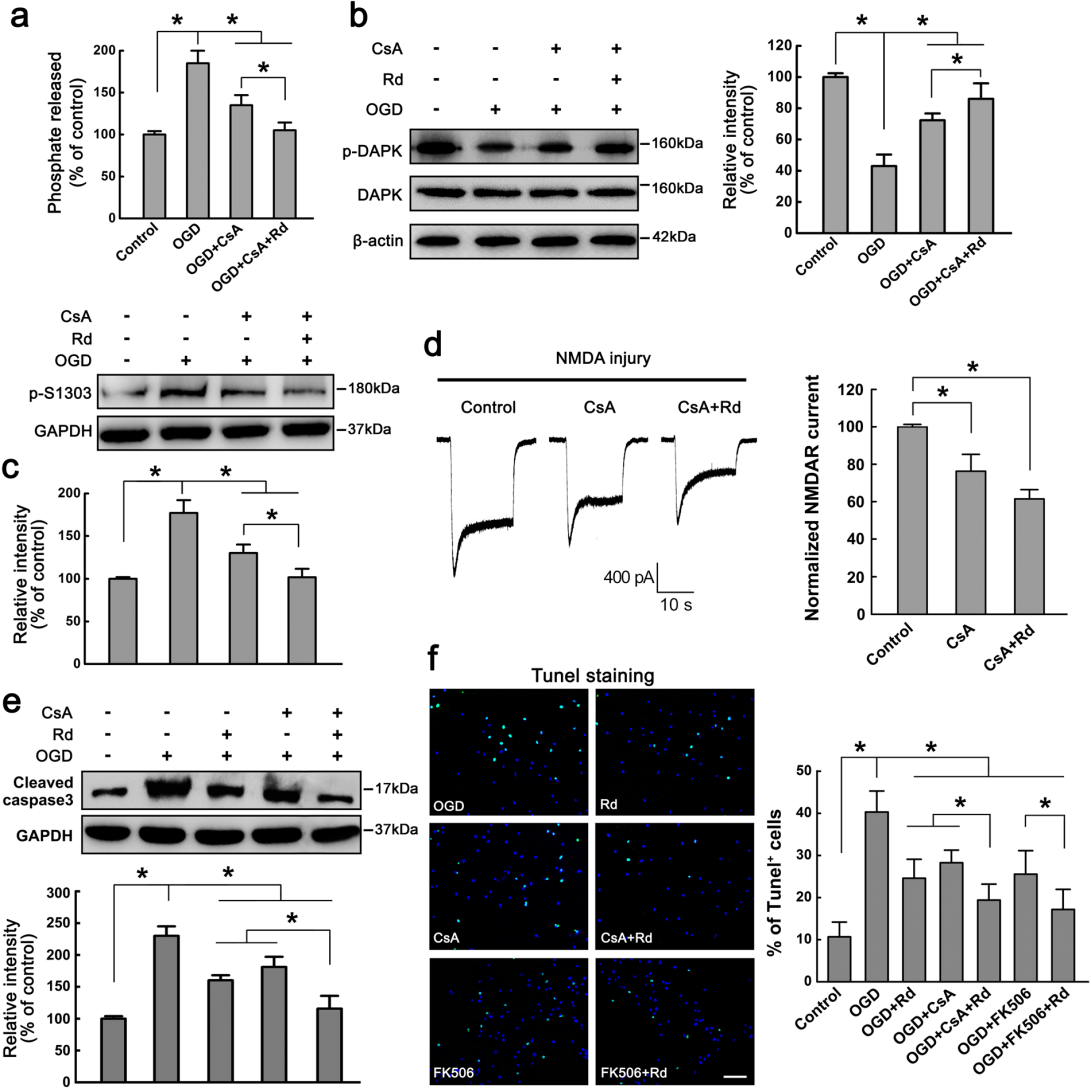
Calcineurin inhibitor CsA mimics Rd to protect against neuronal injury. (**a**) Cell-based enzymatic assay showed that CsA reduced OGD-enhanced calcineurin activity. (b, c) Immunoblotting assay showed that CsA attenuated OGD-induced decreased levels of p-DAPK (**b**) and increased levels of p-Ser1303 (**c**). β-actin and GAPDH were used as internal controls. For comparison easily, shown blots were cropped, and the full-length blots were presented in Supplementary Fig. 10. (**d**) After NMDA injury, CsA reduced NMDAR currents. CsA combined with Rd showed more effective than CsA alone. *, *p* < 0.05. (**e,f**) The changes in the expression cleaved caspase3 (**e**) and the percentages of Tunel^+^ apoptotic cells (Green, **f**) with the treatment of CsA, Rd, CsA plus Rd, FK506, or FK506 plus Rd after cultured neurons were exposed to OGD insult. Total cell numbers revealed by Hoechst 33342 staining (blue) were comparable in each group. Scale bar = 50 μm. Shown blots were cropped to see easy, and the full-length blots were presented in Supplementary Fig. 10. *p < 0.05 vs. the control. Error bars = S.E.M.

To confirm whether Rd-induced inhibition of calcineurin could protect against neuronal injury, we used a blocker of calcineurin cyclosporine A (CsA), to test whether CsA could recur the effects of Rd. The cellular calcineurin activity assay carried out in OGD-injured neurons showed that CsA (10 μM) significantly reduced OGD-enhanced calcineurin activity by 26.7%, and simultaneous addition of Rd decreased enzyme activity by 43.2% (Fig. 7a). Consistently, application of CsA alone or CsA plus Rd also attenuated OGD-induced decreased levels of p-DAPK and increased levels of p-Ser1303 (Fig. 7b,c). Displayed blots were cropped, and full-length blots were presented in Supplementary Fig. 10. After NMDA injury, CsA could reduce NMDA-evoked NMDAR currents from to −1833.6 ± 69.5 pA (n = 20) to −1434b.0 ± 70.6 pA (n = 20), and combination with Rd caused a further reduction of NMDAR currents to −1136.1 ± 84.3 pA (n = 20) (Fig. 7d). After exposure to OGD insult, the expression of cleaved caspase3 and the number of Tunel^+^ apoptotic neurons were markedly elevated compared with the controls. Application of CsA alone or CsA plus Rd decreased OGD-induced increased caspase3 expression and percentages of apoptotic cells (Control: 10.4 ± 4.1%; OGD: 40.2 ± 5.1%; Rd: 25.6 ± 6.6%; CsA: 28.8 ± 5.7%; CsA+Rd: 19.8 ± 3.5%) (Fig. 7e,f). Displayed blots were cropped, and full-length blots were presented in Supplementary Fig. 10. Similar results were also observed in MCAO-injured rats. Administration of Rd (10 mg/kg, i.p.) or/and CsA (10 mg/kg, i.p.) reduced the infract volume of rat brain and ameliorated animal neurological functions significantly (Supplementary Fig. 4).

Considering the fact that apart from its inhibitory effect on calcineurin, CsA is also able to inhibit cyclophilin D, one of components of the mitochondrial permeability transition pore, to exert its cytoprotective effect under appropriate circumstances^51^. To confirm that calcineurin inhibition was involved in the protection afforded by CsA in the OGD-injured model, we employed FK506, a more specific inhibitor of calcineurin, as a control. The results showed that FK506 (10 μM) alone or FK506 plus Rd reduced the percentages of apoptotic cells (FK506: 26.1 ± 7.8%; FK506 + Rd: 17.9 ± 7.6%) as well (Fig. 7f). However, it was noted that the effect of FK506 was comparable to that of CsA or Rd (Fig. 7f), implying that calcineurin inhibition by CsA or Rd may be mainly responsible for the neuroprotection against OGD-induced neuronal injury. Taken together, the results above suggested that calcineurin inhibition could mimic Rd’s effects to protect against NMDAR-mediated neuronal injury.

Under physiological condition, there is a balance between extrasynaptic NMDAR-triggered neuronal death pathway and synaptic NMDAR-mediated neuronal survival pathway^14^. Following ischemic stroke, this balance is broken and neuronal survival pathway, often involving the serine/threonine kinase Akt and extracellular signal-regulated kinase ERK^52,53^, is weaken. We then examined whether Rd acted on neuronal survival pathway in OGD-injured neurons as well. OGD insult significantly decreased the expression levels of p-Akt and p-ERK1/2 as compared with the controls. Treatment of Rd, but not CsA, attenuated OGD-induced decreased levels of p-Akt and p-ERK. No significant differences of total Akt and ERK expression were observed among all these groups (Supplementary Fig. 5). Displayed blots were cropped, and full-length blots were presented in Supplementary Fig. 13. Taken together, our findings suggested that Rd mitigated extrasynaptic NMDAR-triggered neuronal injury by antagonizing DAPK-mediated NR2b phosphorylation as well as enhanced Akt- and ERK-mediated neuronal survival pathways.

## Discussion

In the present study, we demonstrated that Rd, a kind of protopanaxadiol ginsenoside isolated from *Panax ginseng* or *Panax notoginseng*, protected against neuronal excitotoxic injury by inhibiting the activity of calcineurin phosphatase. As a result, calcineurin inhibition resulted in phosphorylation enhancement to cause DAPK inhibition, which decreased NR2b phosphorylation at S1303 and sequential NMDAR channel conductance, ultimately attenuating NMDAR-mediate excitotoxic injury. These results were not consistent with the hypothesis assumed by our previous study^21^ that Rd may serve as a potential NMDAR blocker.

*Panax ginseng* and *Panax Notoginseng* are two traditional herbal medicinal remedies widely used in Asia for more than 2000 years, which are now also popular in the western world. Modern pharmacological research has demonstrated that ginseng and notoginseng are beneficial for cardiovascular and cerebrovascular diseases. Ginsenosides are believed to be the principle active ingredients for ginseng’s efficacy, among which protopanaxadiol ginsenoside-Rd is attracting more and more attentions for its inhibitory effect on ROCC. Consistently, our previous study also revealed that Rd mildly suppressed Ca^2+^ influx possibly by inhibiting NMDAR, one of important ROCCs expressed in neurons^21^. This study further provided *in vitro* and *in vivo* evidence that Rd reduced neuronal NMDAR currents through especially acting on extrasynaptic NR2b subunits (Figs. 1 and 2). Apart from ROCC, voltage-dependent inward Ca^2+^ channel (VDCC) also involves Ca^2+^ influx but it was reported that Rd had little effect on VDCC^26^. By contrast, other ginsenosides, such as Rb1, Rc, Re, Rf, Rg1, Rg3, and Rh2 exhibited inhibitory effects of VDCC more or less^54–56^. All these lines of evidence suggested that Rd may exert its neuroprotective effects by acting on VDCC, such as NMDAR selectively.

In searching of the specific target of Rd on NMDAR, we performed cell transfection and radioligand binding assays. However, these results clearly showed that Rd actually did not bind to the classic sites of NMDAR (Fig. 3). It was noted that other ginsenosides, such as Rb1, Rb2, Rg1, Rg2, Rg3, and Rh2, also showed little affinity to NMDAR. These unexpected results suggested that there must exist other mechanism(s) for Rd to affect NMDAR functions. Phosphorylation is one of crucial mechanisms involved in modulation of NMDAR conductance^45–48^, we examined the effects of Rd on the phosphorylation of NMDAR NR2b subunit. As expected, our results showed that Rd decrease injury-induced increased levels of NR2b phosphorylation at S1303 by inhibiting the activation of DAPK (Figs. 4 and 5), a death-associated protein kinase, which was known for its effect on neuronal cell death and neurodegenerative disease^57^. Further, we revealed that the inhibitive effect of Rd DAPK was mediated by its blockage of calcineurin, a Ca^2+^/calmodulin-dependent serine phosphatase^49,50^. It was noted that even in the absence of calmodulin, which is required for full activity of calcineurin, Rd still exerted an inhibitory effect on calcineurin (Fig. 6). Moreover, we revealed that other ginsenosides (e.g., Rb1, Rb2, Rg1, Rg2, Rg3 or Rh2) inhibited calcineurin to a certain extent as well, whereas Rd showed the most efficient. Overall, the effect of PPD was somewhat higher than that of PPT. Therefore, these data provided evidence that Rd indirectly affected NMDAR functions by changing its phosphorylation state via regulating phosphatase-kinase system negatively. Specifically, Rd inhibited calcineurin phosphatase, and calcineurin inhibition inhibited DAPK kinase, causing reduction of NR2b phosphorylation at S1303 and sequential NMDAR conductance.

In order to confirm that Rd inhibited calcineurin activity to affect NMDAR functions, we compared the effects of Rd with calcineurin inhibitor CsA. As expected, in addition to its ability to inhibit calcineurin and DAPK activities and sequential NR2b phosphorylation at Ser1303, CsA also showed obvious neuroprotective effects, such as reducing harmful NMDAR current, attenuating OGD-induced apoptosis *in vitro*, decreasing animal infract volumes, and ameliorating behavioral performance (Fig. 7). These results were consistent with other studies^58,59^, in which CsA, initially served as an immunosuppressant in transplant medicine, was found to have neuroprotective effects probably due to its ability to block mitochondrial permeability transition pore activation and preserve normal mitochondrial function^58^. The present study provided the supplemental evidence that CsA could also exert the neuroprotective effects by affecting NMDAR functions via a phosphorylation way. More importantly, we demonstrated that the neuroprotective effects of Rd were comparable to, even slightly higher than (without significance) that of CsA while combined Rd and CsA treatment exerted more prominent neuroprotection against neuronal injuries than either Rd or CsA alone. We proposed that this superposed effect might owe to the evidence that Rd inhibited calcineurin activity not dependent entirely on calmodulin. Additionally, it was worthwhile to note that apart from its inhibitory effects on NMDAR-elicited neuronal cell death, Rd also enhanced the activities of Akt and ERK, two important signaling pathways involved in neuronal survival after ischemic insults^52,53^. However, in our study CsA seemingly showed less significant effects on Akt and ERK activities.

A large number of studies have shown that ginsenosides could have promising preventive and therapeutic efficacy on experimental or clinical stroke damage. In terms of potential neuroprotective mechanisms of ginsenosides, though not yet fully understood, the majority opinion is that ginsenosides exert their neuroprotective effects through multiple ways, including anti-oxidant, anti-inflammation, anti-apoptosis, anti-autophagy, neurogenesis, and others^60,61^. Nevertheless, as far as the early stage of ischemic stroke is concerned, one of key events responsible for neuronal death is the excitotoxicity triggered by excitatory amino acids (e.g., glutamate). That is, excessive glutamate results in overactivation of NMDA receptor (a kind of ROCC), which induces cytosolic Ca^2+^ overload and then triggers a cascade of molecular events, such as formation of reactive oxygen species, lipid peroxidation, mitochondrial dysfunction, calpain and caspase activation, etc. For this reason, many studies have been carried out to explore whether ginsenosides act on ROCC or Ca^2+^-associated VDCC, the upstream effectors triggering excitotoxicity. Previous and our studies have showed that ginsenosides Rb1, Rd, Rh2, and Rg3 were found to attenuate glutamate- and NMDA-induced neurotoxicity or to inhibit NMDAR currents in cultured neurons^21,62^. These findings suggested that ginsenosides may act on NMDAR to exert their neuroprotective effects. However, the present result (Fig. 3f) and other study^62^ revealed a very low affinity of ginsenosides to NMDAR or other receptor ligand-gated ion channels, indicating an indirect role of ginsenosides in affecting ROCC. To test this proposal, here we showed that Rd affected NMDAR functions indirectly by acting on calcineurin. Therefore, ginsenosides could be beneficial for the prevention or treatment of ischemic stroke through regulating multipronged mechanisms, but in the early or acute stage, a calcineurin-mediated inhibitory effect on NMDAR-dependent excitotoxicity may be at least one of crucial mechanisms.

Taken together, inconsistent with our previous hypothesis that Rd may serve as an NMDAR blocker, the present evidence demonstrated that Rd may inhibit NMDAR functions in an indirect way, especially after ischemic insults. Specifically, Rd could inhibit calcineurin and sequential DAPK activities, causing the decrease of NR2b phosphorylation and NMDAR conductance, ultimately protecting against NMDAR-mediated excitotoxic injury.

## Supplementary information

Supplementary information.
