# Supplementary material for: Protopanaxadiol ginsenoside Rd protects against NMDA receptor-mediated excitotoxicity by attenuating calcineurin-regulated DAPK1 activity
Source: Sci Rep. 2020 May 15;10:8078. doi: 10.1038/s41598-020-64738-2 (PMC7228936; doi:10.1038/s41598-020-64738-2)

Protopanaxadiol ginsenoside Rd protects against NMDA receptor-mediated excitotoxicity by attenuating calcineurin-regulated DAPK1 activity

Chen Zhang[1,2*], Xuedong Liu[1*], Hui Xu[3*], Gengyao Hu[1], Xiao Zhang[1], Zhen Xie[1], Dongyun Feng[1], Rui Wu[1], Gang Zhao[1] and Ming Shi[1]

[1] Department of Neurology, Xijing Hospital, Fourth Military Medical University, Xi'an, China

[2] Department of Neurology, Second Artillery General Hospital of PLA, Beijing, China

[3] Institute of Neurosciences, Fourth Military Medical University, Xi'an, China

[*] These authors contributed equally to this work

Corresponding authors: Gang Zhao and Ming Shi, Department of Neurology, Xijing Hospital, Fourth Military Medical University, 15 Changle-Xi Road, Xi'an 710032, Shaanxi province, China; TEL: +86 29 8477 5368, Fax: +86 29 8255 1806. Email: biomidas@163.com

Competing interests: The authors have no conflicts of interest to declare.

Supplementary Table 1. Ginsenosides for radioligand binding assay

Chemicals	Formula	MW	Purity (%)	Classification
Ginsenoside-Rb1	$C_{54}H_{92}O_{23}$	1109.29	98.9	PPD
Ginsenoside-Rb2	$C_{53}H_{90}O_{22}$	1079.27	98.8	PPD
Ginsenoside-Rd	$C_{48}H_{82}O_{19}$	963.15	98.4	PPD
Ginsenoside-Rg1	$C_{42}H_{72}O_{14}$	801.01	98.3	PPT
Ginsenoside-Rg2	$C_{42}H_{72}O_{13}$	785.01	100.0	PPT
Ginsenoside-Rg3	$C_{42}H_{72}O_{13}$	785.02	100.0	PPD
Ginsenoside-Rh2	$C_{36}H_{62}O_{8}$	622.87	100.0	PPD

MW: Molecular Weight

Supplementary Figure 1. The changes in expression of NR1, NR2a and NR2b after NMDA treatment. GAPDH was used as an internal control. Shown blots were cropped to see easy, and the full-length blots were presented in Supplementary Figure 11. Error bars=S.E.M.

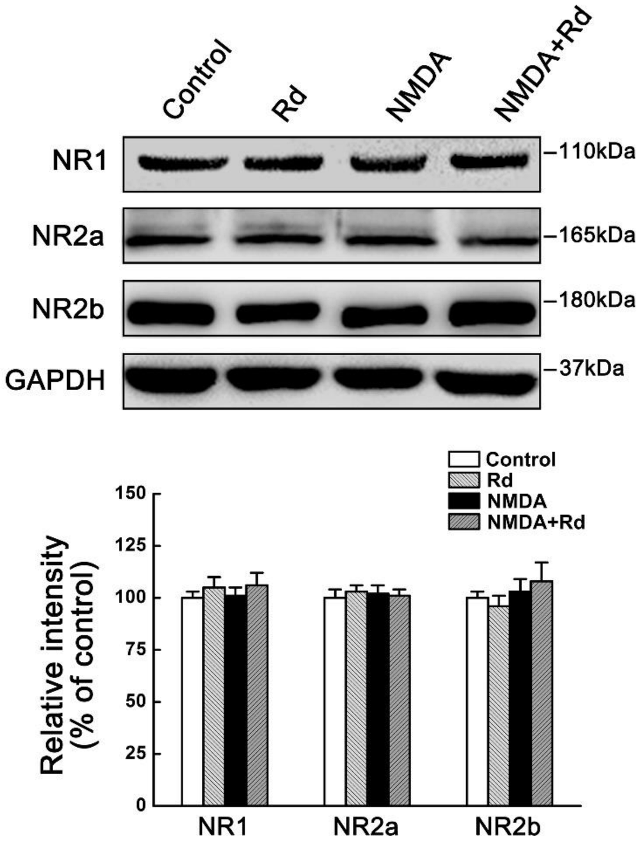

Supplementary Figure 2. The effects of CK59 or Gö6983 on Rd-induced decreased NMDAR currents in NMDA-injured neurons. CK59 (a) or Gö6983 (b) did not block inhibitory effects of Rd on NMDAR currents. *, *p*<0.05 vs. the control or antagonist alone. Error bars=S.E.M.

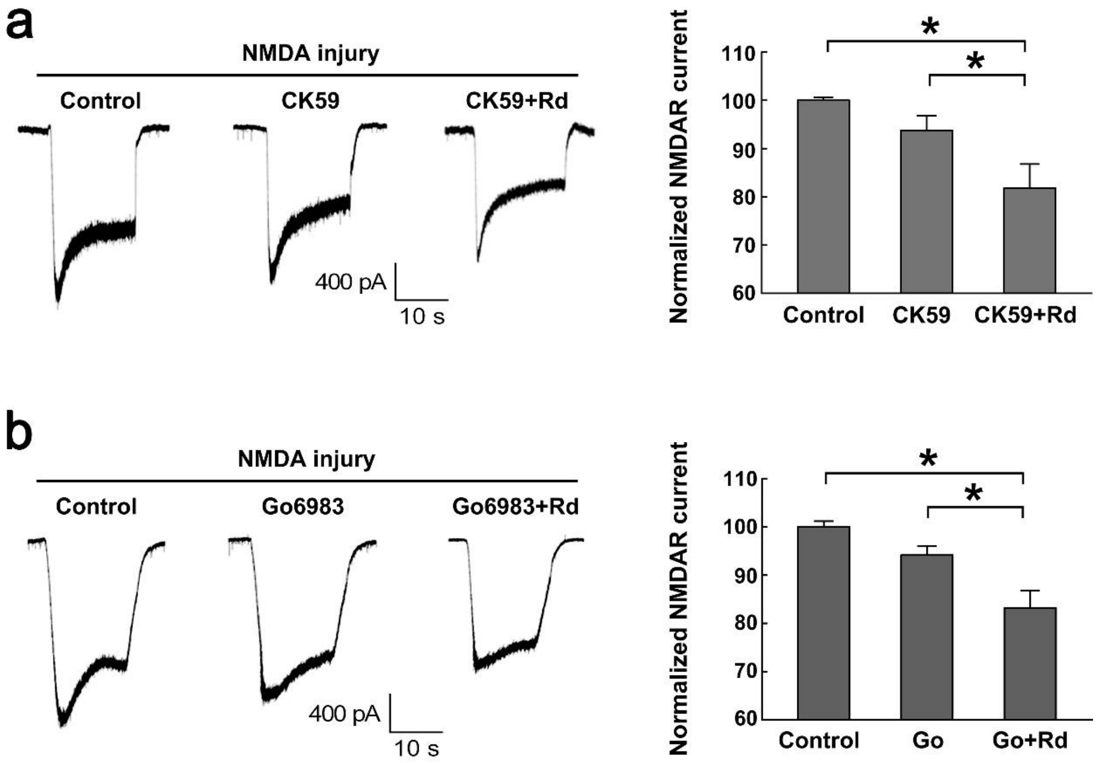

Supplementary Figure 3. The effects of Rd on DAPK phosphorylation in MCAO-injured rat brains. Western blotting results showed that MCAO insult decreased the levels of p-DAPK, which was increased by Rd treatment. β-actin was used as an internal control. Shown blots were cropped to see easy, and the full-length blots were presented in Supplementary Figure 12. #, p<0.05 vs. the control; *, p<0.05 vs. MCAO group. Error bars=S.E.M.

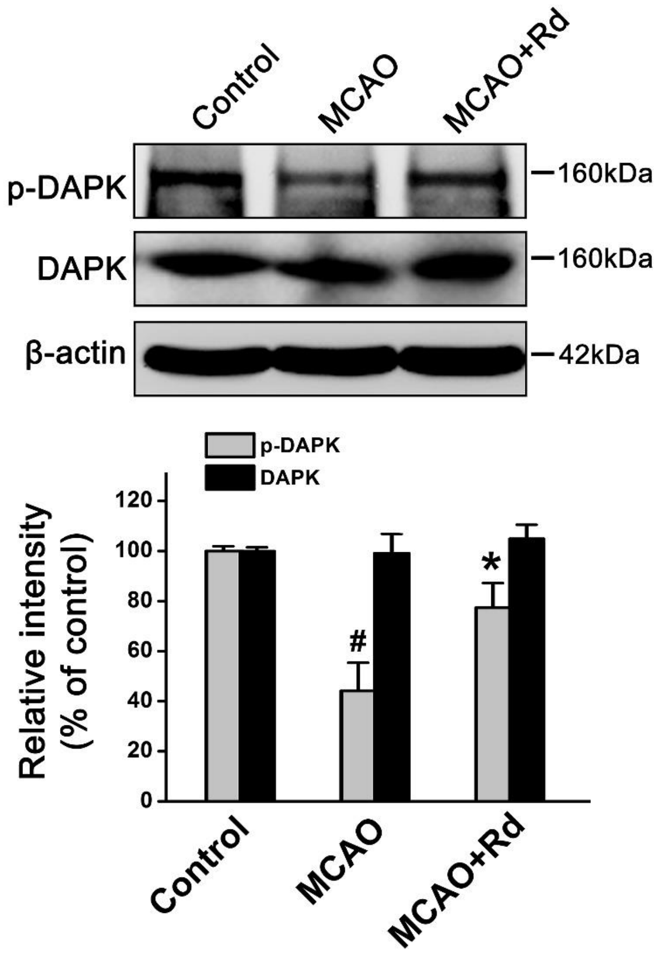

Supplementary Figure 4. CsA mimics Rd to protect against MCAO injury in rats. At 24h post-MCAO, administration of Rd (10 mg/kg, n=6) or/and CsA (10 mg/kg, n=6) reduced the infract volume of rat brain, revealed by TTC stain (a), and improved rat neurological functions (b). *, $p<0.05$ vs. MCAO group (n=6). Error bars=S.E.M.

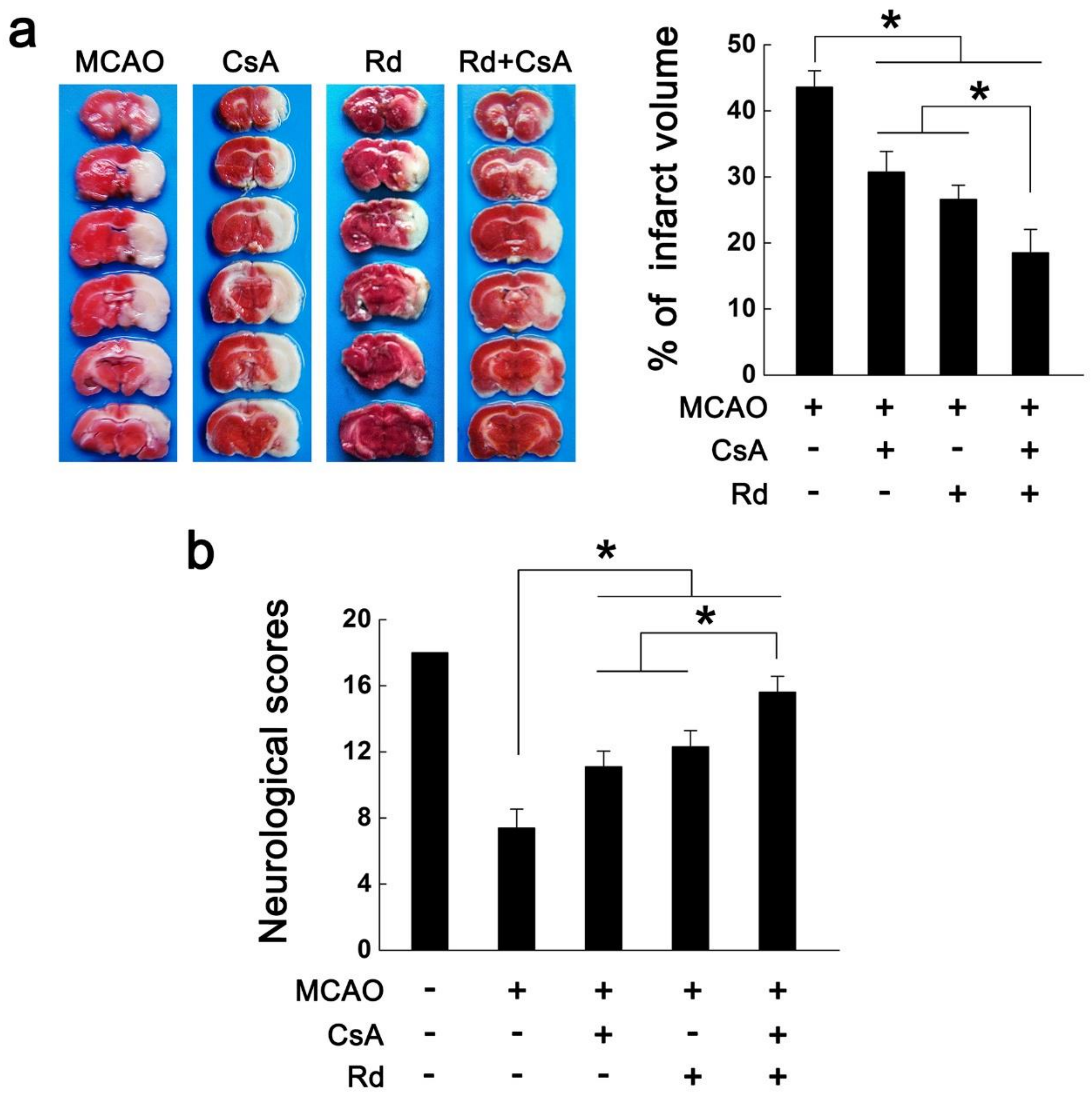

Supplementary Figure 5. Rd promotes neuronal survival pathways. Western blotting analysis showed the changes in the expression of total and phosphorylated Akt (a) and ERK1/2 (b) with the treatment of Rd or CsA after OGD injury. GAPDH was used as an internal control. Shown blots were cropped to see easy, and the full-length blots were presented in Supplementary Figure 13. *, $p<0.05$. Error bars=S.E.M.

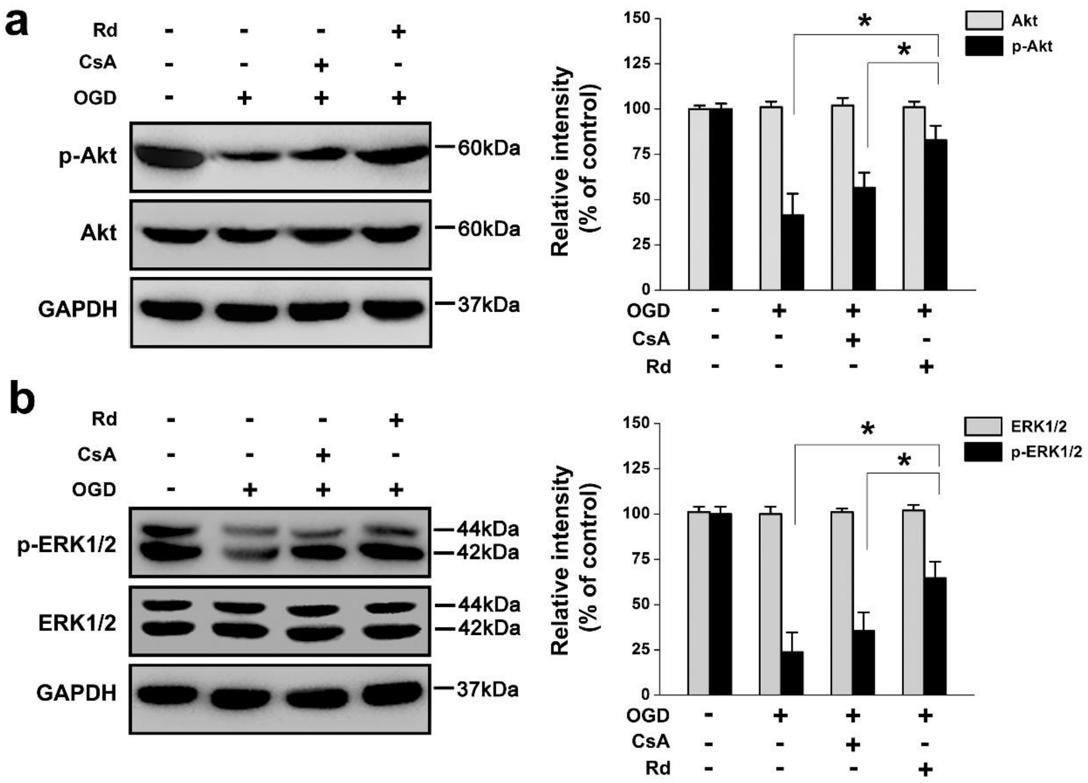

Supplementary Figure 6. Full-length blots for Figure 3(a)

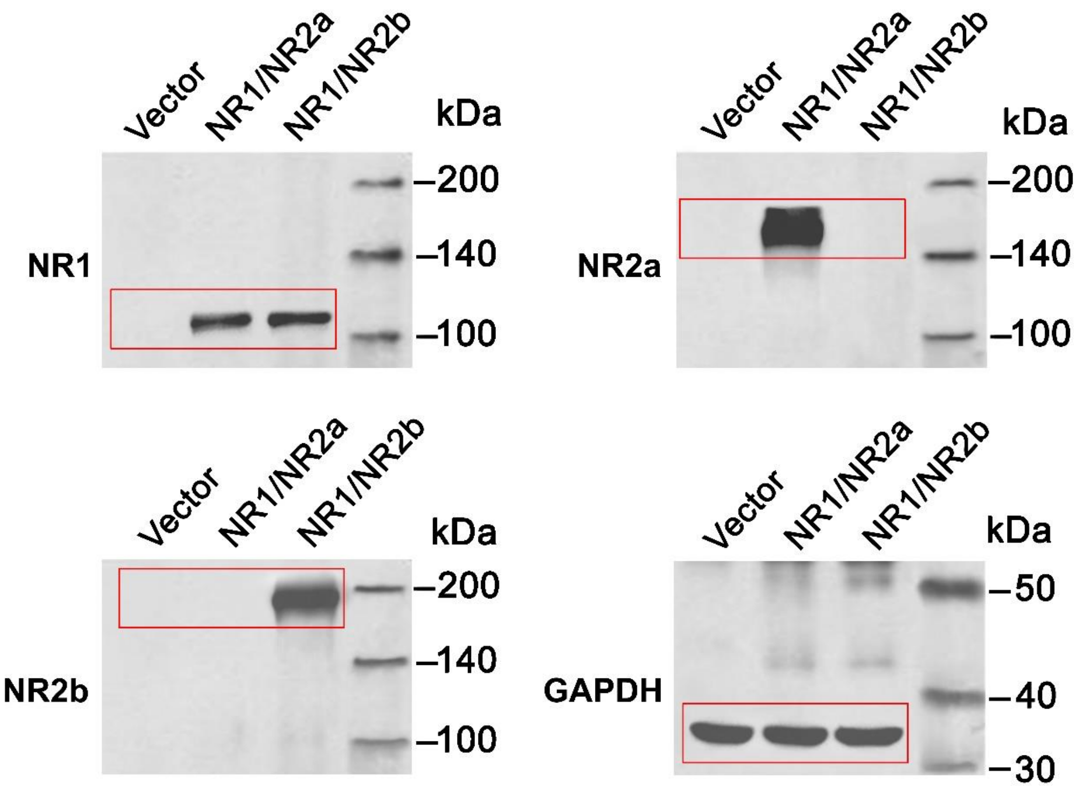

Supplementary Figure 7. Full-length blots for Figure 4(a,b,c)

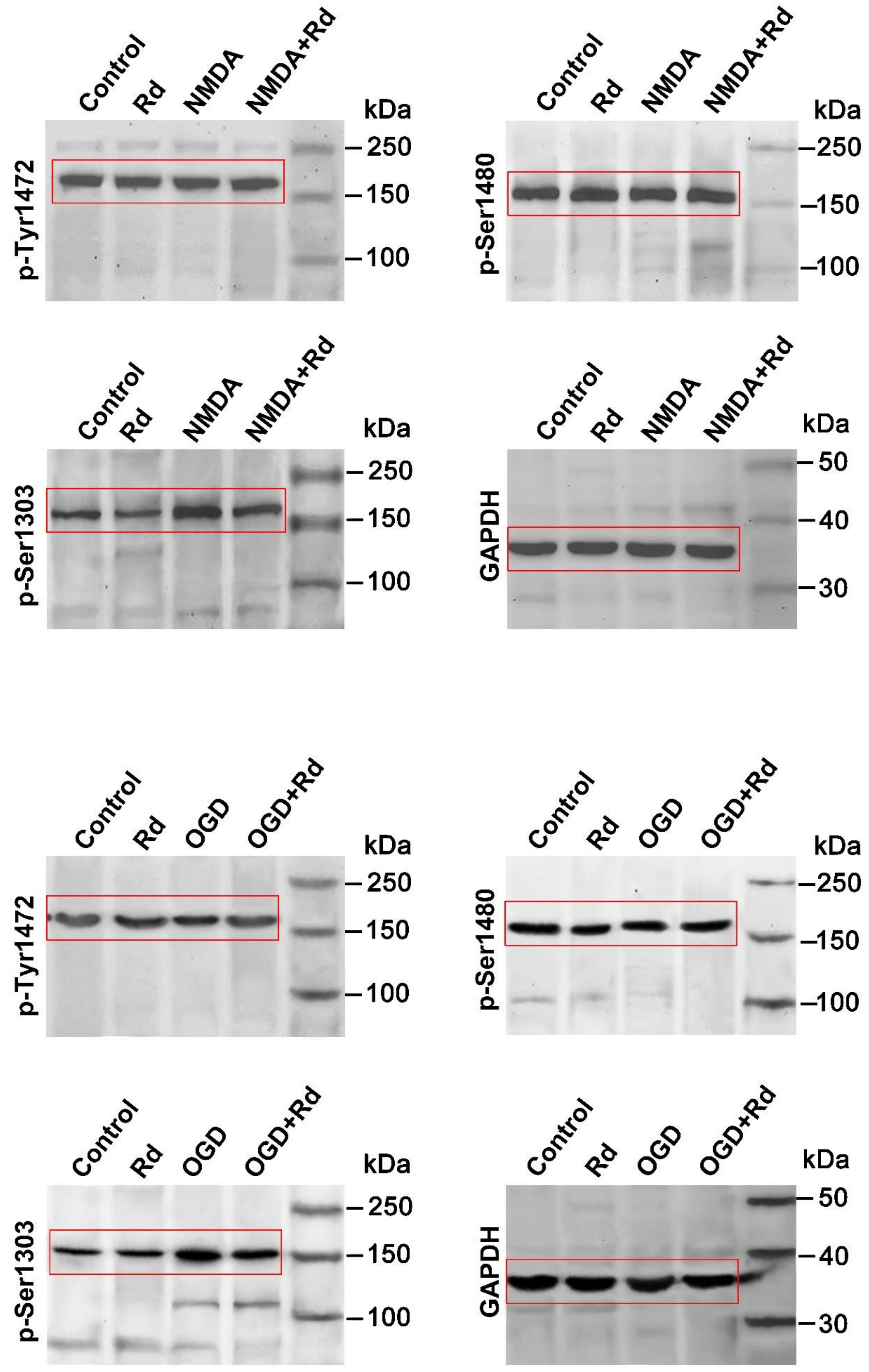

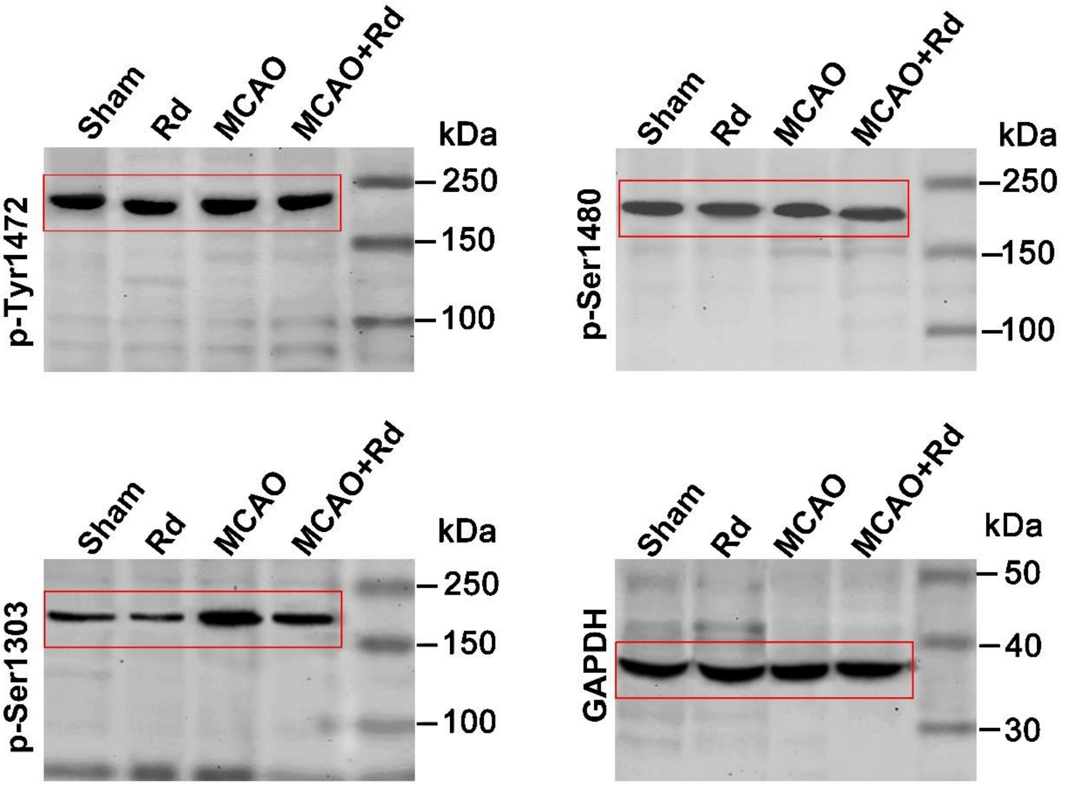

Sham
Rd
MCAO
MCAO+Rd
kDa
p-Tyr1472
250
150
100
p-Ser1480
250
150
100
p-Ser1303
250
150
100
GAPDH
50
40
30

Supplementary Figure 8. Full-length blots for Figure 5(a,d,e)

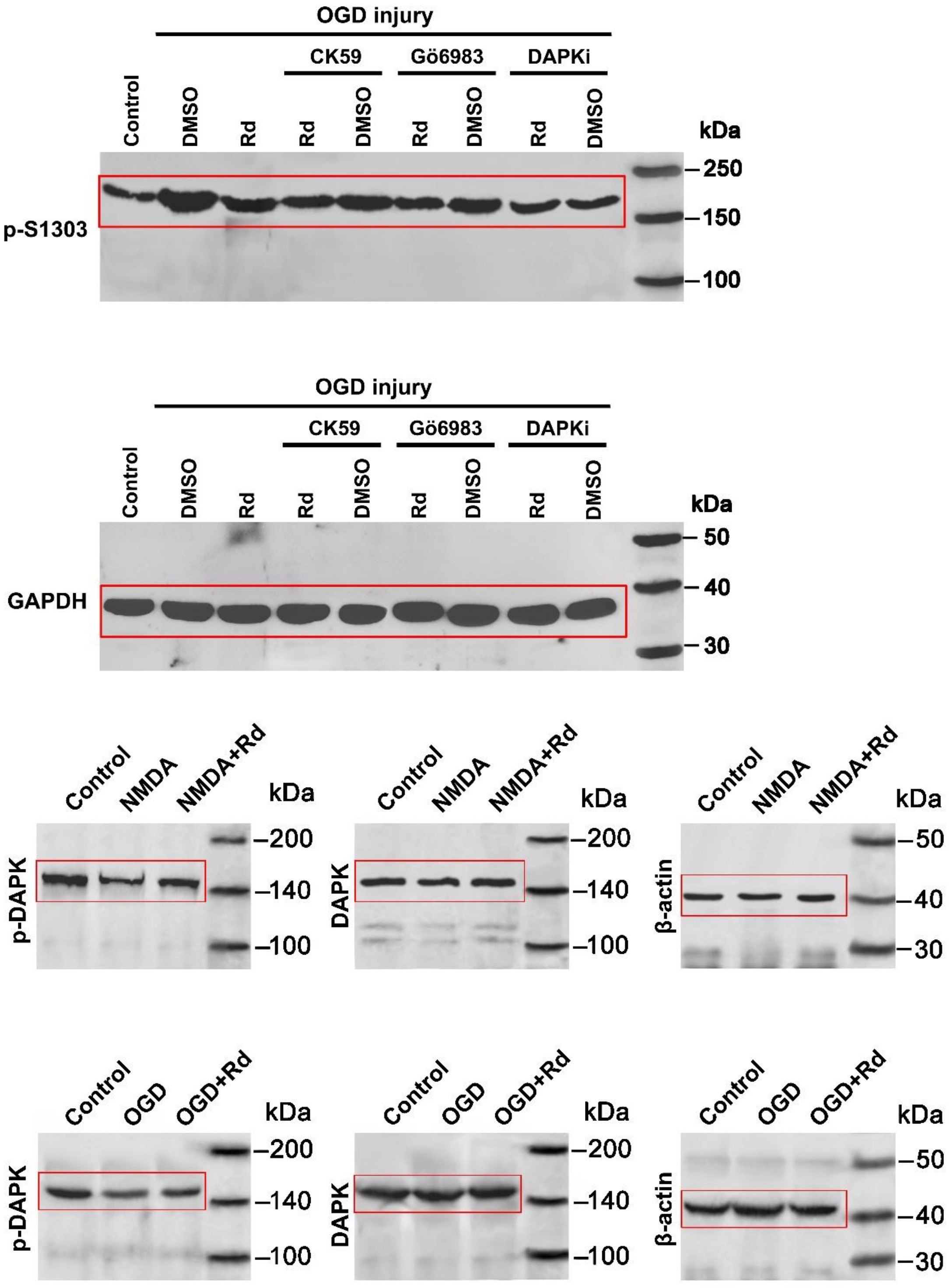

Supplementary Figure 9. Full-length blots for Figure 6(a)

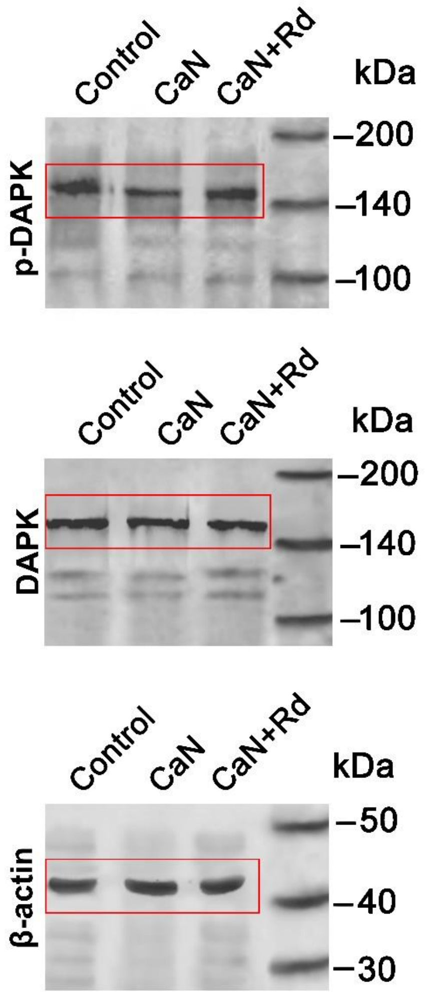

Supplementary Figure 10. Full-length blots for Figure 7(b,c,e)

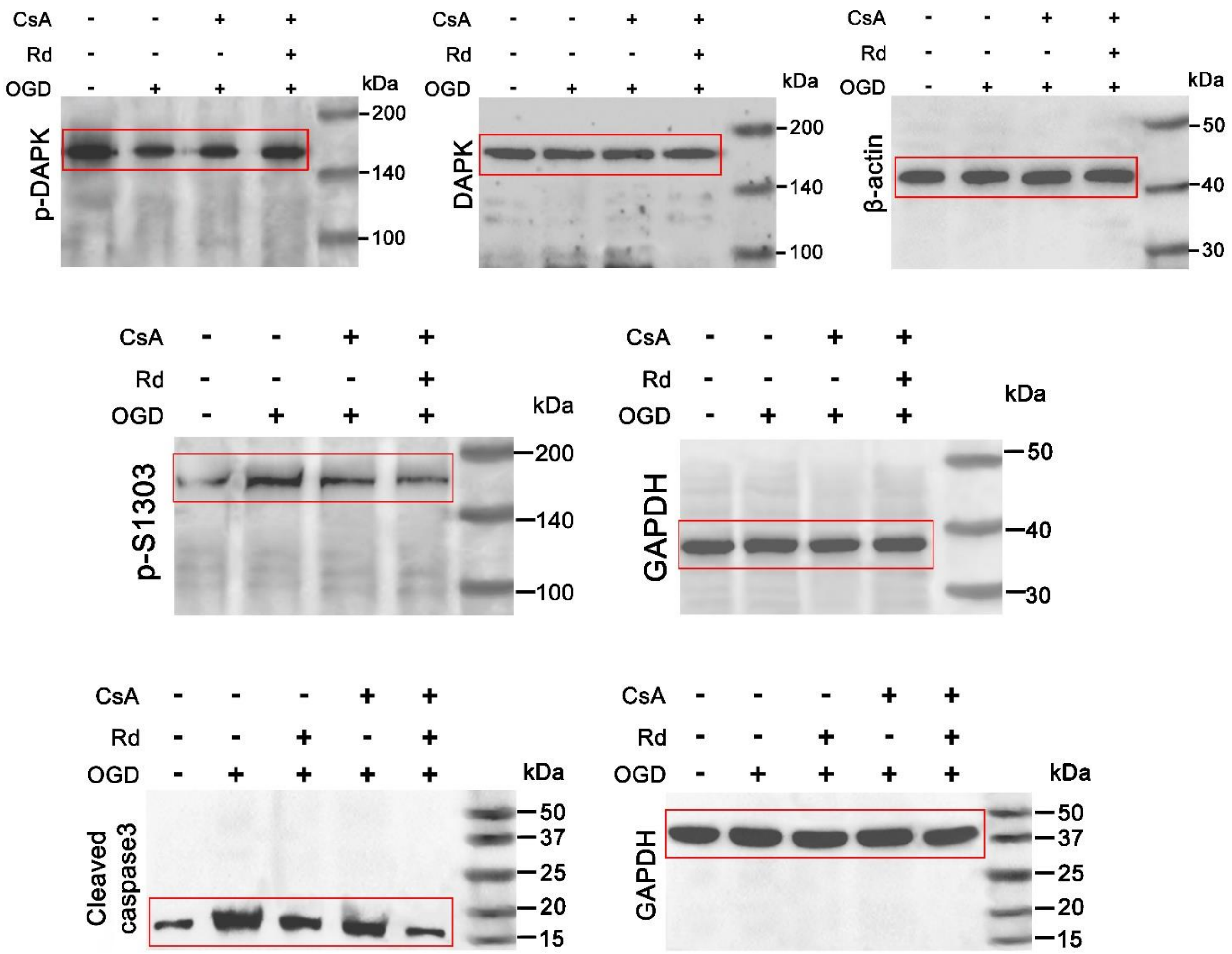

Supplementary Figure 11. Full-length blots for Supplementary Figure 1

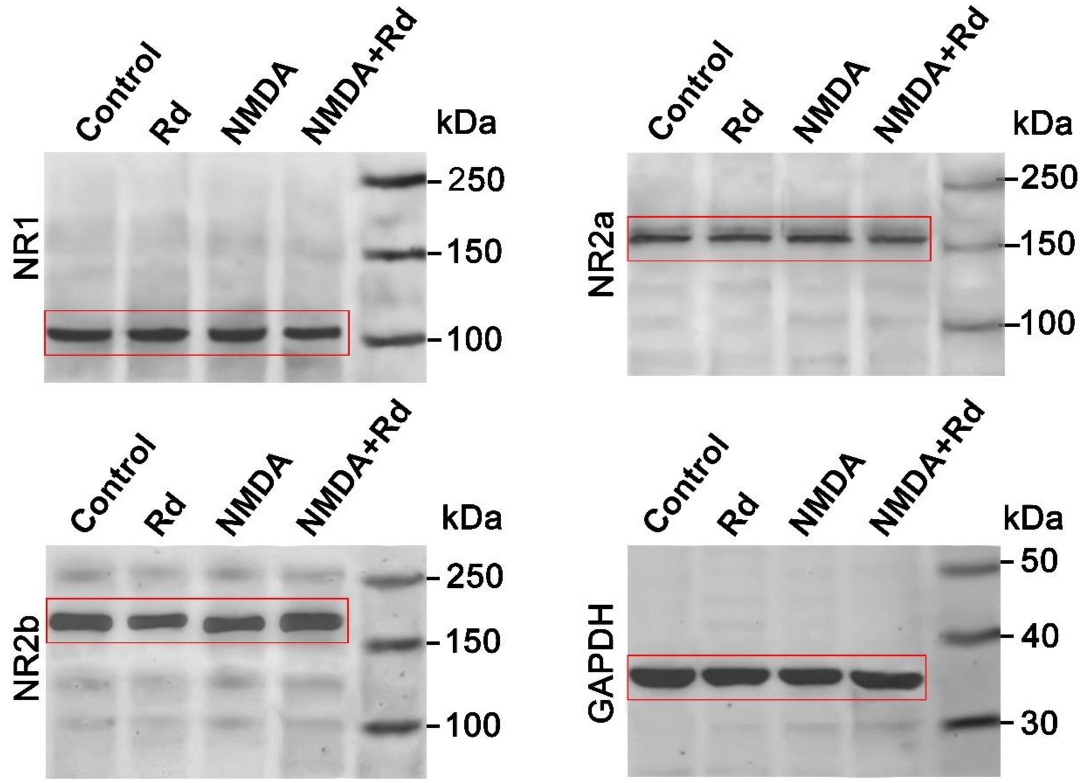

Supplementary Figure 12. Full-length blots for Supplementary Figure 3

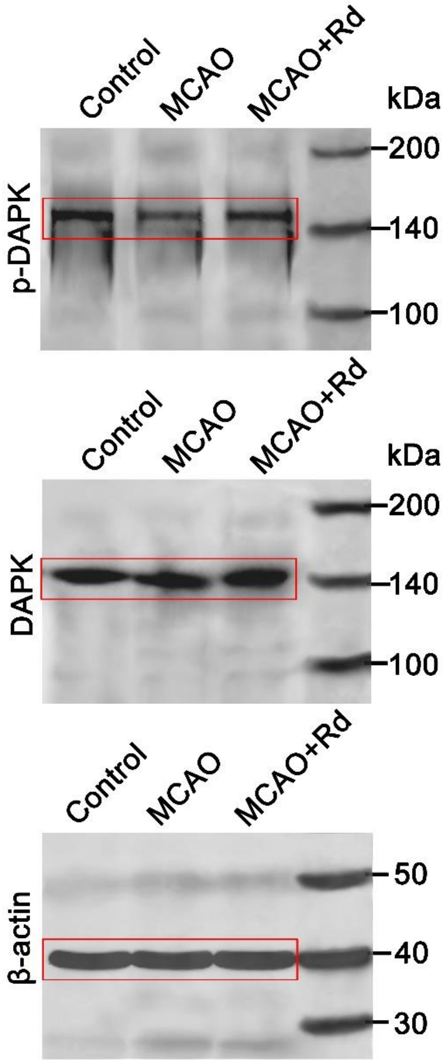

Supplementary Figure 13. Full-length blots for Supplementary Figure 5(a,b)

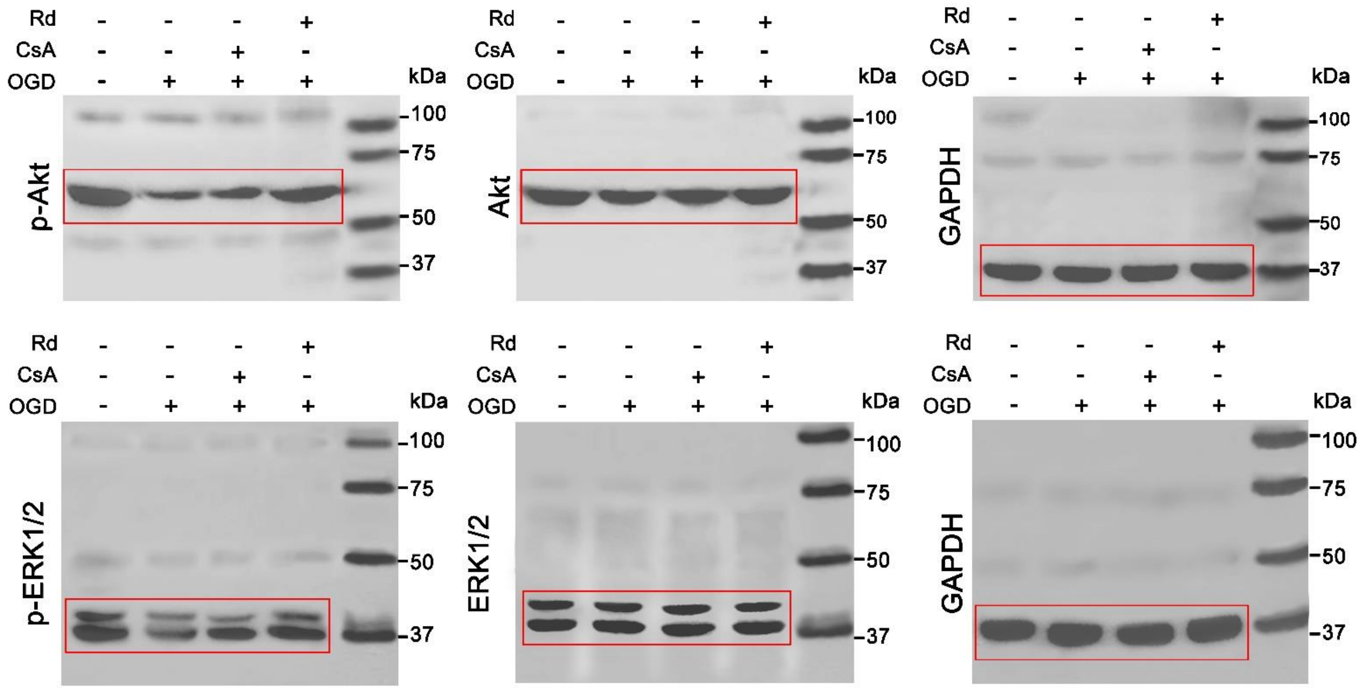

Supplement: Supplementary file 1 — Supplementary information. [file 41598_2020_64738_MOESM1_ESM.pdf]
